# Evaluating the contribution of gut microbiome to the variance of porcine serum glucose and lipid concentration

**DOI:** 10.1038/s41598-017-15044-x

**Published:** 2017-11-02

**Authors:** Xiaochang Huang, Shaoming Fang, Hui Yang, Jun Gao, Maozhang He, Shanlin Ke, Yuanzhang Zhao, Congying Chen, Lusheng Huang

**Affiliations:** State Key Laboratory of Pig Genetic Improvement and Production Technology, Jiangxi Agricultural University, 330045 Nanchang, China

## Abstract

Serum glucose and lipids are important indicators for host metabolic condition. Interaction of host and gut microbes regulates the metabolism process. However, how much the gut microbiome contributes to the variance of serum glucose and lipids is largely unknown. Here we carried out a 16S rRNA gene based association study between cecum microbiome and the concentration of serum glucose and lipids in 240 Chinese Erhualian pigs. We identified tens of bacterial taxa associated with serum glucose and lipids. The butyrate-producing bacteria were significantly associated with serum glucose level. The pathogenic bacteria belonging to *Proteobacteria* and *Fusobacteria* showed significant associations with increased serum lipid levels, while the bacteria *Lactobacillus* and *Bacilli* had negative correlations with serum lipids. Cross-validation analysis revealed that 23.8% variation of serum glucose and 1.6%~6.0% variations of serum lipids were explained by gut microbiome. Furthermore, predicted function capacities related to nutrition intake, transport and carbohydrate metabolism were significantly associated with serum glucose level, while the pathways related to antioxidant metabolism and bile synthesis tended to be associated with serum lipid level. The results provide meaningful information to get insight into the effect of gut microbiome on serum glucose and lipid levels in pigs.

## Introduction

Serum glucose and lipids are important indicators for the condition of body metabolism. Abnormal concentrations of blood glucose and lipids are associated with various kinds of chronic diseases, such as obesity, diabetes as well as cardiovascular diseases. Substantial studies have revealed that both blood glucose and lipid levels are under control of genetics at some extent, and many associated-genes and SNPs were identified^1–4^. However, these genetic variants together account for only ~25–30% of phenotypic variation of blood glucose and lipids^5^, which suggests that a big puzzle remains to be explored.

Mammalian gut microbiota is a complex ecosystem and composed of thousands of heterogeneous microbial species^6^. Recent decade, researches on gut microbiome have depicted important roles of commensal microbial community in carbohydrate digestion, immune system development and disease resistance^7,8^. To date, there are several studies focusing on the relationship between gut microbiome and blood glucose and lipids in humans. Sepp *et al*.^9^ reported that reduction of the proportion of anaerobes in the gut microbiota is significantly associated with a higher blood glucose level and body mass index in elderly people. Kovatcheva-Datchary *et al*. suggested that increased abundance of *Prevotella* is associated with dietary fiber-induced improvement in glucose metabolism. Fu *et al*.^10^ revealed that gut microbiome contributes a substantial proportion of variation in serum lipids. However, the comprehensive knowledge about how gut microbiome affects serum glucose and lipid levels is still absence. Some studies have suggested that short chain fatty acids (SCFAs) produced by fermentation of gut bacteria play important roles in regulating serum glucose and lipids, e.g. Todesco *et al*.^11^ reported an effect of propionate on lowering blood glucose and altering lipid metabolism in healthy subjects; The study in butyrate administrated sheep found that butyrate has an significant effect on blood glucose level, but this effect is strongly associated with the body initial blood glucose^12^. Kristina *et al*.^13^ inferred that the LPS, bile acid, SCFAs, gut hormones and branched-chain amino acids are potential factors linking to glucose dysbiosis.

Pigs have been used as the biomedical model of human diseases for decades because of the similarity of physiological characteristics and digestive system with humans^14^. Compared to mice that are the most popular biomedical model, pigs showed the more similarity of gut microbiome with humans^15^. Of the functional pathways of gut microbiome found in the human catalogue, 96% are present in the pig catalogue^15^. Furthermore, pigs are always raised in a uniformed farm condition and fed the similar formula diet. This suggests that pigs should be a perfect model for studying the contribution of gut microbiome to the variance of blood glucose and lipids. However, to our knowledge, no such study has been reported in pigs.

In the current study, we investigated the microbial composition of cecum lumen samples using 16S rRNA gene sequencing^16^ in a Chinese Erhualian pig population, which was comprised of both castrated boars and gilts, and showed different concentrations of serum glucose and lipids. We evaluated the contribution of gut microbiome to the variance of serum glucose and lipids. We identified tens of bacterial taxa and predicted KEGG function terms of gut microbiome showing significant associations with serum glucose and lipids. The results provided meaningful information about the relationship of homeostasis of serum glucose and lipids to gut microbiome.

## Results

### Microbial diversity of porcine cecum luminal samples from experimental pigs

After quality control, pair-end clean reads were merged into tags. We obtained a total of 4,338,951 tags for all tested samples with an average of 18,162 tags per sample, and ranging from 9,678 to 31,188 tags. At the 97% similarity, we got a total of 2,038 operational taxonomic units (OTUs) in the 240 samples. After rarefaction, the average OTU number for the experimental pigs was 524, ranging from 163 to 683. We observed a significantly higher OTU richness in the castrated boars than that in the gilts (${\rm{P}}=2.62\times {10}^{-6}$, *t-*test) (Supplement Fig. 1a). The castrated boars also had a higher shannon’s index than gilts (*P* = 1.70 × 10^−4^, *t-*test). Similar to gut microbial composition in humans, *Bacteroidetes* (51.97 ± 15.39%) and *Firmicutes* (28.76 ± 14.18%) were the two most abundant taxa, followed by *Spirochaetes* (Supplement Fig. 1b).

### Association of gut bacteria with the variance of serum glucose and lipids

We found that the phenotypic values of both serum glucose and lipids followed the normal distribution (Shapiro test *P* value > 0.05). We performed the two-tailed *t-*test to evaluate the effect of gender on traits, and found the significant influence of sex on the values of serum low density lipoprotein cholesterol (LDL) level, ratio of low density lipoprotein cholesterol to low density lipoprotein cholesterol (LDL/HDL) and atherosclerosis index (AI) (Table 1). We first evaluated the association of microbial richness and diversity with serum glucose and lipids. Both OTU number and alpha-diversity indexes were not significantly associated with serum glucose or lipids. We then performed the association analysis between the relative abundance of OTUs or taxa and the concentration of serum glucose and lipids. At false discovery rate (FDR) < 0.05 (*P* = 3.5 × 10^−3^), we identified 179 OTUs significantly associated with serum glucose. Among these 179 OTUs, 77 were annotated to *Lachnospiraceae*, 25 OTUs to *Ruminococcaceae*, 13 OTUs to *Bacteroides* and 9 OTUs to *Prarprebotellaceae*. We observed that most of the OTUs annotated to *Lachnospiraceae* and *Ruminococcaceae* were positively associated with serum glucose, while the OTUs annotated to *Bacteroidetes*, *Prevotella* and *Fusobacteria* showed negative association with serum glucose (Supplement Table 1). We also identified 15 OTUs significantly associated with total cholesterol (TC), 73 OTUs with LDL/HDL, and 62 OTUs with AI, while we did not identify any OTUs associated with HDL, triglyceride (TG) and LDL at FDR < 0.05 (Supplement Tables 2,3 and 4). Of the 150 OTUs associated with serum lipids, only 4 OTUs were shared by TC, AI and LDL/HDL, 45 OTUs were shared by AI and LDL/HDL, whereas the other 101 OTUs were specifically associated with each trait. We found that 38 associations (25.3%) were detected by quantitative analysis, 41 associations (27.3%) were identified by binary analysis and 71 associations (47.3%) were detected by meta-analysis of binary and quantitative analysis. The OTU307 assigned to the *Treponema* showed the most significant association with TC (*P* = 1.51 × 10^−7^), whereas, the OTU30 annotated to *Bacteroidetes* and the OTU862 to *Fusobacteriaceae* had the most significant association with LDL/HDL and AI, respectively (*P* = 6.05 × 10^−8^ and 4.11 × 10^−7^).

**Table 1:** Summary of the phenotypic values of serum glucose and lipids in Erhualian pigs.

	Castrated boars	Gilts	Total	Comparison between boars and gilts (P value)
	n = 113	n = 127	n = 240	
GLU	3.25 ± 2.12	2.86 ± 1.67	3.05 ± 1.86	0.11
TCHOL	2.24 ± 0.36	2.18 ± 0.37	2.21 ± 0.36	0.18
TG	0.21 ± 0.13	0.22 ± 0.12	0.22 ± 0.12	0.62
HDL-C	1.16 ± 0.40	1.23 ± 0.40	1.19 ± 0.41	0.18
LDL-C	1.57 ± 0.26	1.40 ± 0.32	1.48 ± 0.31	4.6E-06
LDL/HDL	1.58 ± 0.54	1.27 ± 0.55	1.42 ± 0.65	2.5E-04
AI	1.24 ± 0.74	0.96 ± 0.68	1.09 ± 0.81	8.7E-03

**Figure 1:**
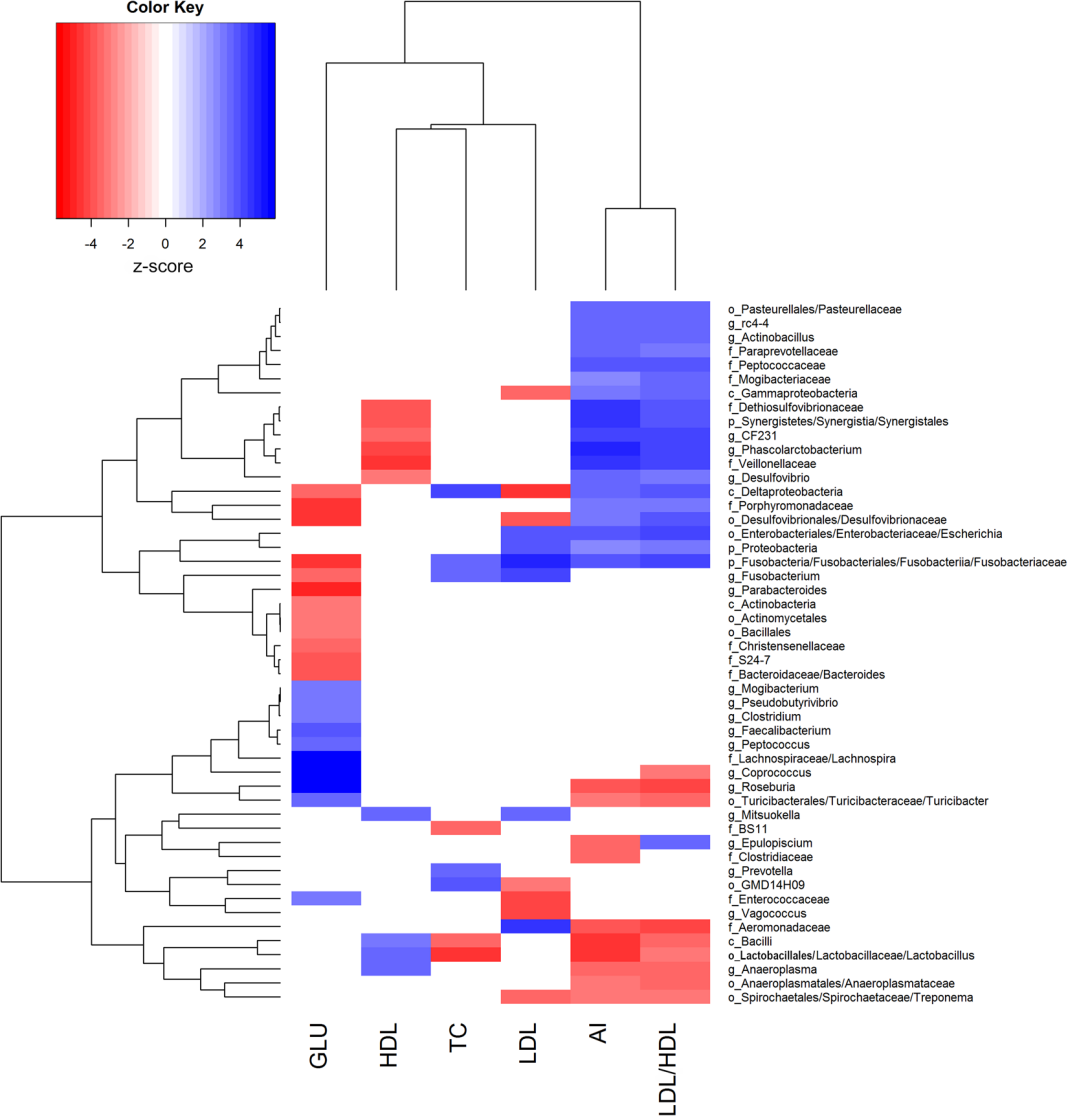
Heatmap of microbial taxa significantly associated with serum glucose and lipids. The associated *P* values were converted to normal Z score. P-phylum, c-class, o-order, f-family and g-genus.

At the taxonomic level, we identified 24 taxonomies significantly associated with blood glucose (GLU) (FDR < 0.05), including 11 taxonomies positively associated with the increased serum glucose (Fig. 1). We found that most of the positively associated taxonomies belong to *Firmicutes* that can digest dietary polysaccharide (Supplement Table 5). Among them, the butyrate-producing bacteria *Coprococcus* ^17^ (*P* = 4.11 × 10^−9^), *Roseburia* ^18^ (*P* = 7.75 × 10^−9^), *Lachnospiraceae* ^17^ (*P* = 2.42 × 10^−8^), *Faecalibacterium* ^19^ (*P* = 1.79 × 10^−4^) and *Clostridium* ^20^ (*P* = 1.63 × 10^−3^) showed the strong association with GLU. We also found 13 taxa showing significantly negative association with GLU. Most of these bacteria belong to *Bacteroidetes* and *Proteobacteria*, such as *Parabacteroides* (*P* = 1.56 × 10^−6^), *Bacteroides* (*P* = 6.08 × 10^−5^), *Porphyromonadaceae* (*P* = 3.21 × 10^−5^), and *Desulfovibrionales* (*P* = 3.51 × 10^−6^) (Supplement Table 5). We further identified the positive correlation between ratio of *Firmicutes* to *bacteroidetes* and serum glucose level (*P* = 4.61 × 10^−4^).

We identified a total of 89 significant associations related to 28 unique taxonomies for serum lipid traits, including 8 associations for TC, 10 for HDL, 13 for LDL, 29 for LDL/HDL and 29 for AI, while no association was identified for TG. Most of these serum lipid-associated taxonomies belong to *Proteobacteria* and *Fusobacteria*. The *Deltaproteobacteria* showed the strongest positive association with TC (*P* = 1.11 × 10^−5^), *Lactobacillus* had the strongest positive association with HDL (*P* = 3.07 × 10^−5^), and *Fusobacteriaceae* was most significantly associated with LDL (*P* = 6.82 × 10^−7^), whereas *Lactobacillaceae* showed the most negative association with TC (*P* = 2.34 × 10^−6^), *Veillonellaceae* with HDL (*P* = 1.01 × 10^−5^) and *Deltaproteobacteria* with LDL (*P* = 6.91 × 10^−6^) (Fig. 1 and Supplement Tables 6~10). We noticed that some of these significant associations were shared among traits. For example, sulfate-reducing bacteria *Deltaproteobacteria* and *Desulfovibrionaceae* were positively associated with TC (*P* = 1.11 × 10^−5^), LDL/HDL (*P* = 1.02 × 10^−4^ and 7.60 × 10^−5^) and AI (*P* = 2.50 × 10^−4^ and 9.95 × 10^−4^), but negatively associated with HDL (*P* = 1.65 × 10^−4^ and 1.16 × 10^−3^). Interestingly, some of the serum lipid-associated taxa have been reported to associate obesity or cardiac vascular disease (CVD). For instances, *Phascolarctobacterium* is positively associated with obesity^21^, and *Veillonella* has been supposed to be a possible causal bacteria in atherosclerosis^22^. In this study, both *Phascolarctobacterium* and *Veillonellaceae* were positively associated with LDL/HDL (*P* = 1.70 × 10^−5^ and 4.74 × 10^−5^) and AI (*P* = 1.14 × 10^−6^ and 2.56 × 10^−6^), but negatively associated with HDL (*P* = 1.54 × 10^−5^ and 1.01 × 10^−5^). We also identified some potential bacteria that were correlated to the lower cholesterol level, e.g. *Bacilli* and *Lactobacillus* were negatively associated with TC (*P* = 5.84 × 10^−4^ and 2.34 × 10^−6^), LDL/HDL (*P* = 5.78 × 10^−4^ and 1.25 × 10^−3^) and AI (*P* = 5.17 × 10^−6^ and 5.51 × 10^−6^); and *Treponema* was negatively associated with LDL (*P* = 1.31 × 10^−3^), LDL/HDL (*P* = 1.76 × 10^−3^) and AI (*P* = 1.76 × 10^−3^).

### Association of predicted function capacities of gut microbiome with serum glucose and lipids

To link the potential function capacity of gut microbiome to the level of serum glucose and lipids, we performed functional prediction of gut microbiome based on the 16S rRNA gene sequences. The associations between the predicted KEGG pathways and the levels of serum glucose and lipids were estimated. We identified a total of 95 pathways that were significantly associated with serum glucose at FDR < 0.05 (Supplement Table 11). Among these 95 pathways, 27 pathways showed positive associations with GLU, most of which are related to nutrient intake and nutrition sensing (such as flagellar assembly, bacterial motility protein, transporters and ABC transporters), carbohydrate metabolism (e.g. pentose phosphate pathway, starch and sucrose metabolism, and fructose and mannose metabolism), and lipid metabolism (bile secretion and glycerolipids metabolism). Notably, the insulin signaling pathway was positively associated with serum glucose level (*P* = 2.15 × 10^−4^). We also identified a total of 68 pathways negatively associated with GLU, including amino acid metabolism, metabolisms of cofactors and vitamins as well as biosynthesis of secondary metabolites.

**Figure 2:**
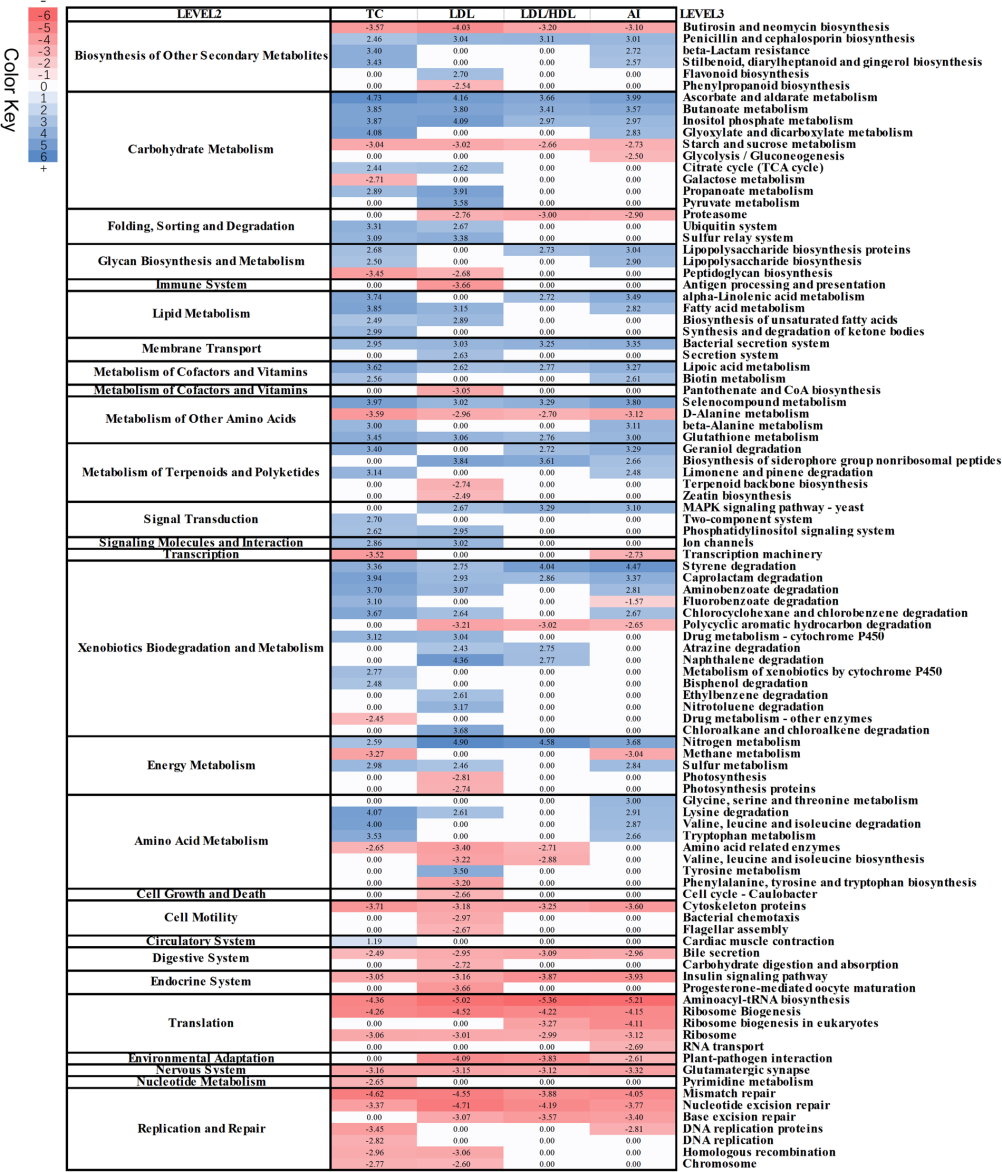
Heatmap of predicted KEGG pathways significantly associated with serum lipids. The associated *T* values were used for plot.

**Figure 3:**
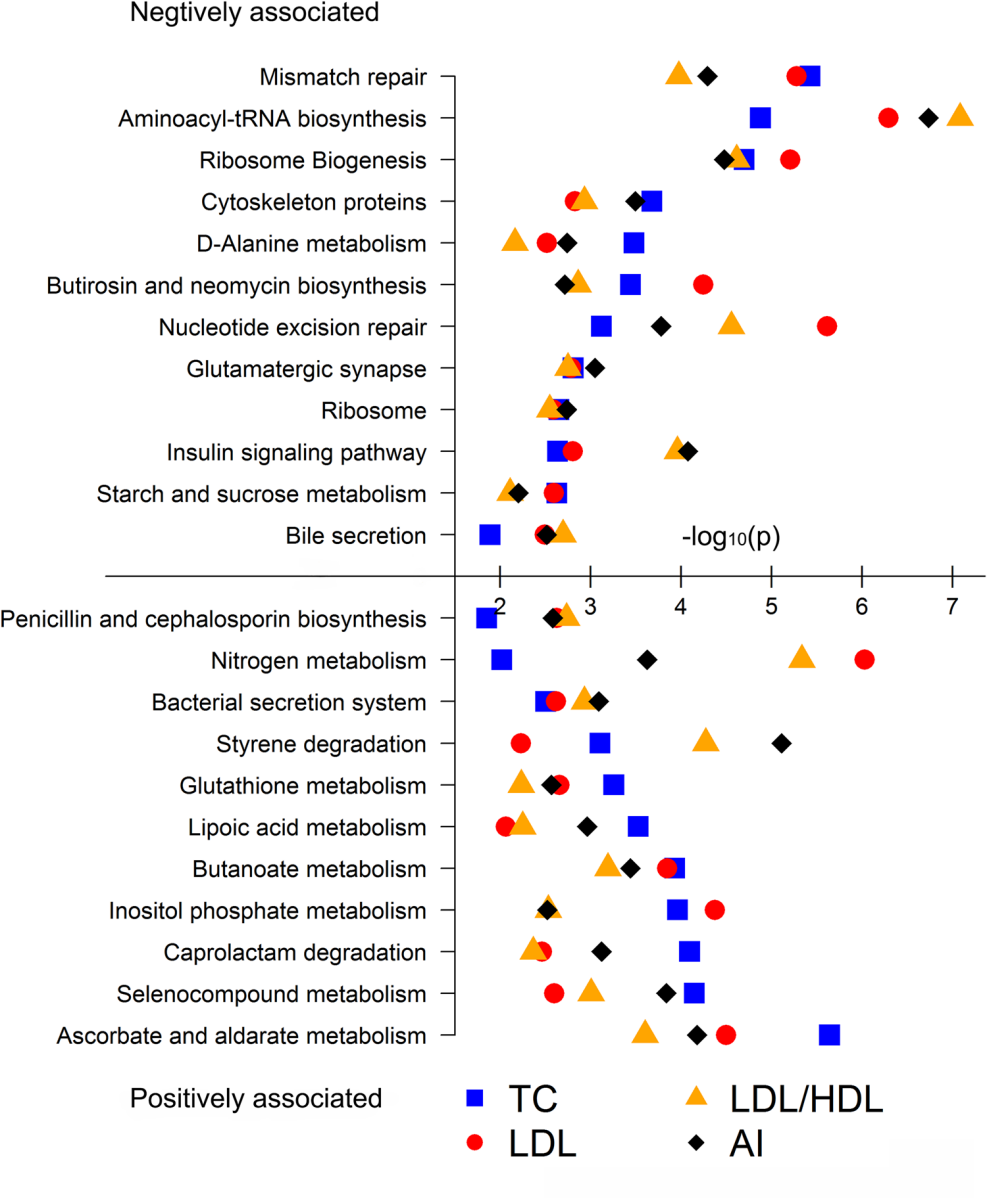
The associated KEGG pathways shared by LDL/HDL, AI, LDL and TC. The X- axis indicates the –log10 *P* values obtained in association analysis.

At FDR < 0.05, we identified 65, 68, 38 and 54 KEGG functional pathways significantly associated with TC, LDL, LDL/HDL and AI, respectively (Fig. 2). The pathways involved in lipid metabolism, metabolisms of cofactors and vitamins, and xenobiotics biodegradation showed positive associations with serum lipids, while translation (such as biosynthesis of aminoacyl-tRNA and ribosome), replication and repair, and cell motility (such as cytoskeleton proteins and flagellar assembly) were negatively associated with serum lipid traits. We identified 23 serum lipid-associated KEGG pathways shared by TC, LDL, LDL/HDL and AI (Fig. 3). Eleven out of these shared pathways were positively associated with the four traits. Interestingly, four antioxidant metabolism related pathways were positively associated with LDL, TC, LDL/HDL and AI (Fig. 3). These antioxidants have been reported to play important roles in protecting body from suffering atherosclerosis and coronary heart disease^23^. Moreover, we identified 12 pathways showing negative association with TC, LDL, LDL/HDL and AI (Fig. 2), including bile secretion, starch and sucrose metabolism, and insulin signaling pathway. However, we did not identify any KEGG pathways significantly associated with HDL.

### Contribution of gut microbiome to the variation of serum glucose and lipids

**Figure 4:**
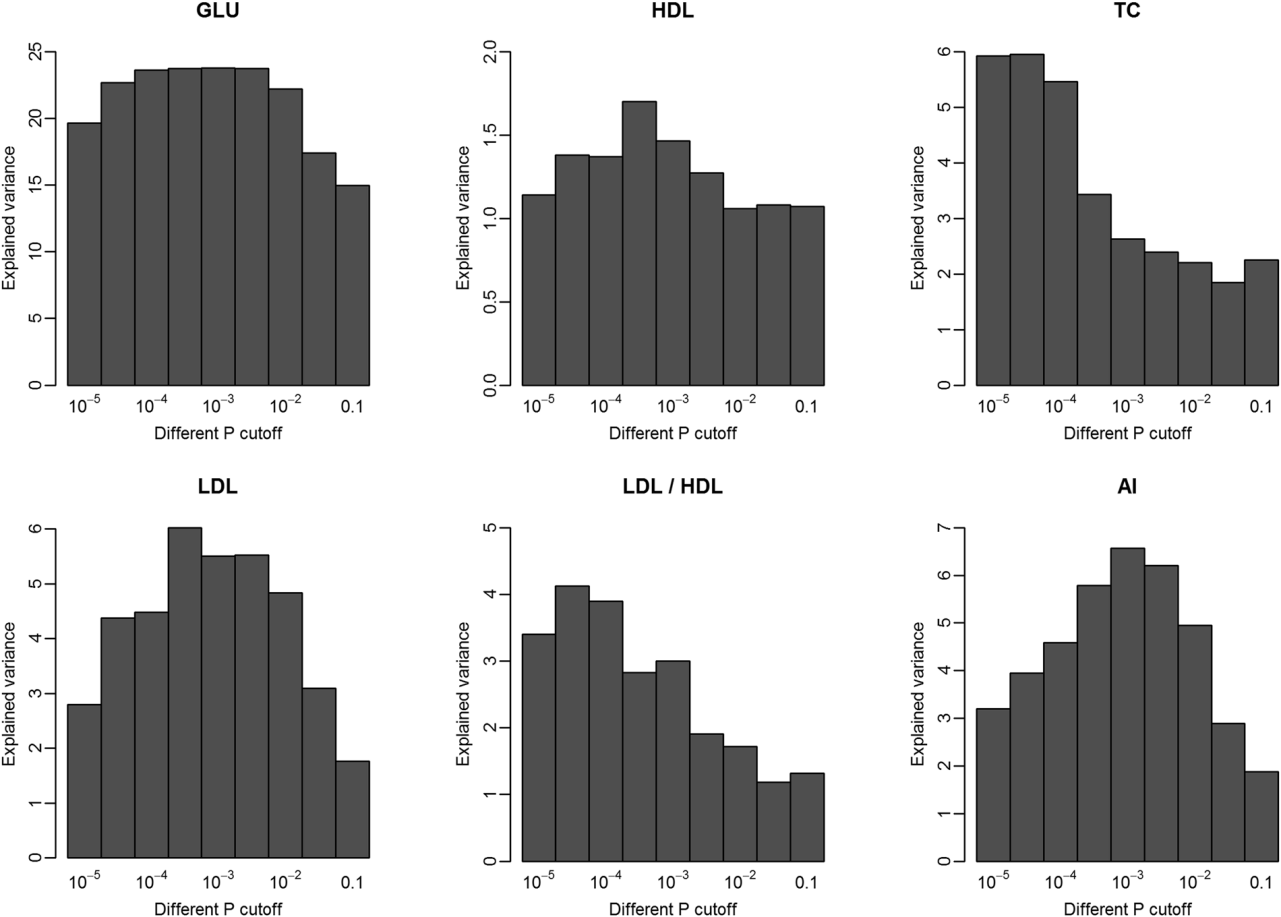
The variation of serum glucose and lipids explained by gut microbiome at different significance level.

We further determined the contribution of gut microbiome to the variation of serum glucose and lipids by the 100× cross-validation analysis at different *P* value threshold (ranging from 10^−5^ to 0.1) as described by Fu *et al*.^10^. The OTUs identified at *P* = 1.0 × 10^−5^ level in the discovery set explained 19.63% variation in GLU in the validation set, 1.14% in HDL, 5.92% in TC, 2.79% in LDL, 3.4% in LDL/HDL, and 3.19% in AI. When the association cutoff of *P* value was increased and more OTUs were included in the model, the explained variation increased to 23.78% in GLU, 5.95% variation in TC, 6.02% in LDL, 4.12% in LDL/HDL, 6.56% in AI, and 1.69% in HDL (Fig. 4).

## Discussion

Normal concentrations of serum glucose and lipids are important to human health. Gut microbiota may be involved in the regulation of serum glucose and lipid levels because it plays important roles in host metabolism^10^. In this study, we identified tens of OTUs and bacterial taxa significantly associated with porcine serum glucose and lipid levels, and found that gut microbiome has a substantial contribution to the variation of porcine serum glucose and lipid levels. The result was consistent with the report in humans, in which gut microbiome contributes to a substantial proportion of the variation in human blood lipids^10^.

Unlike the study in humans, we used the cecum luminal samples for investigating the association between gut microbiome and serum glucose and lipid levels. This should be due to the reason of the great abundance and diversity of microbiota in the cecum. Both our previous study and the report by Looft *et al*. suggested that the cecum has the higher abundance and diversity of microbiota than small intestine^3,16^. Furthermore, microbiota in stool is a mix of shed mucosal bacteria, and most from the colon and lumenal microbes^24^. In addition, previous studies have suggested that SCFAs play important roles in the regulation of blood glucose and lipids^11,12^. As we have known, cecum is a major fermentation location where SCFAs are produced through fermenting the diet fibers by bacteria.

In this study, we revealed the strong association between butyrate producing bacteria (such as *Coprococcus*, *Lachnospiraceae*, *Roseburia* and *Faecalibacterium prausnitzii*) and the increased serum glucose level. This result was consistent with the finding reported by Gorka *et al*.^25^, which showed that newborn calves fed with the high butyrate diet had a higher serum glucose level. The study in sheep also indicated that butyrate injection increased blood glucose level in individuals with a low initial blood glucose^12^. Furthermore, in fasting animals with a low blood glucose, butyrate may play a role in promoting glucogenesis^26^. Considering the functions of butyrate in recovery of intestinal mucosa and morphology, and promoting effectiveness of digestion and absorption of sucrose^27,28^, we speculated that butyrate producing bacteria may play an important role in regulating serum glucose level through carbohydrate fermentation products, such as butyrate, which improve the intestinal health, and promote the digestion and absorption. *Prevotella* and several pathogenic or opportunistic pathogenic bacteria were identified to negatively associate serum glucose level. In humans, *Prevotella copri* was the main species driving the association between biosynthesis of branched-chain amino acids and insulin resistance^29^. This result implied that the bacteria inducing inflammation may affect serum glucose level. Sandrine *et al*.^30^ observed that the OTUs from *Parabacteroide* and *Actinobacteria* were negatively associated with serum glucose level in the microbial colonization process. In this study, we also identified that *Actinobacteria* and *Parabacteroide* were negatively associated with porcine serum glucose level (*P* = 1.42 × 10^−3^ and 1.56 × 10^−6^).

Most of the bacteria positively associated with LDL/HDL and AI belong to *Proteobacteria* and *Fusobacteria*, and are pathogenic bacteria or opportunistic pathogen, such as *Desulfovibrio*, *Fusobacteriaceae* and *Enterobacteriaceae*. These bacteria have been reported to associate inflammation^31,32^, indicating the relationship between inflammation-related bacteria and the serum LDL/HDL and AI level. Inflammation always accompanies with the high levels of blood lipids^33,34^. In our previous study, we also found that *Escherichia fergusonii* and *Escherichia coli* from *Enterobacteriaceae* were enriched in the pigs with high fatness^35^, suggesting that inflammation-related bacteria not only increase fat deposition, but also affect blood lipids. *Lactobacillus* showed a negative association with serum lipid level in this study. Consistent with this result, the bacterial strains from *Lactobacillus* can significantly decrease serum TC and LDL in the rats fed with high cholesterol diet^36^. *Lactobacillus* has been reported to be a major source of bile salt hydrolase activity^37^. Bile acid activity of commensal bacteria interplaying with host hepatic enzymes together promotes digestion and absorption of dietary lipids^38^. Functional prediction of cecum microbiome in this study also implied that the increased abundance of bile secretion might associate the decreased level of porcine serum lipids. In addition, the genus *Bacilli* was also identified to negatively associate TC, LDL/HDL and AI, but positively associate HDL. Strains from *Bacilli* have been reported to produce fibrinolytic enzyme which possesses unique property to degrade fibrin blood clots^39,40^.

We identified tens of the predicted function terms that were correlated with serum glucose/lipid levels, which implied that cecum microbiome may have the potential function capacity influencing the blood glucose/lipid. For instance, the pathways related to antioxidant metabolism and bile secretion tended to be associated with porcine serum lipid level. However, metagenomic prediction only provides a reference, but not a precise reflection for microbial function. Metagenomic sequencing analysis would be needed to further elucidate how the cecum microbiome affects serum glucose and lipid levels. We investigated the contribution of gut microbiome to the variation of serum glucose and lipids. Similar to the results reported by Fu *et al*.^10^, we found that gut microbiome contributes a substantial proportion of the variations of serum glucose and lipids. Fu *et al*.^10^ found that gut microbiome showed less contributions to the variation of TC and LDL. However, in this study, cecum microbiome contributes less to the variations of TG and HDL, but more to the variations of TC and LDL. This should be due to the different species of experimental subjects (human *vs*. pigs), diet and the sampling site for microbiome analysis (cecum *vs*. feces).

## Conclusion

We found that the butyrate-producing bacteria should be an important regulator for serum glucose level, which increase the normal serum glucose level through improving the intestinal health, and promoting the digestion and absorption. We also identified tens of bacteria significantly associated with serum lipid level. Most of serum lipid-associated microbial taxa belong to the pathogenic bacteria, suggesting that inflammation induced by pathogenic bacteria increases serum lipids. Cross-validation analysis found that gut microbiome contributes to a substantial proportion of the variations of LDL/HDL, AI, LDL, TC and GLU. This observation provides an important insight into the role of gut microbiome in regulating serum glucose and lipids. The results also suggested that gut microbiome should be an important target for regulating serum glucose and lipids, and for therapy of cardiovascular diseases.

## Methods

### Experimental pig cohort and sample collection

A total of 240 Chinese Erhualian pigs were used in this study, including 127 gilts and 113 castrated boars. All experimental pigs were raised in a fattening house which was comprised of 30 pens. Each pen housed about 10 pigs with mixture of gilts and castrated boars. All pigs were provided the corn-soybean formula diet including 16% of crude protein, 3100kj of digestible energy and 0.78% of lysine two times a day. Water was available ad libitum from nipple drinkers. The boars were castrated at the age of 60 days. All experimental pigs were slaughtered at the age of 300 days after fasting but water free overnight as described previously^3^. The experimental pigs were healthy and did not receive any treatment of antibiotics within at least 2 months before slaughter. Arterial blood was collected to a pro-coagulation tube during the bloodletting process. All cecum lumen samples were harvested in the 7-ml sterile tubes within 30 min after slaughter and immerged into the liquid nitrogen immediately. After transported to laboratory, the lumen samples were transferred into −80 °C freezer until use. All animal works were performed according to the guidelines for the care and use of experimental animals established by the Ministry of Agriculture of China. This study was specifically approved by Animal Care and Use Committee (ACUC) in Jiangxi Agricultural University.

### Measurement of serum glucose and lipids

Serum was isolated from whole blood at a centrifugal speed of 3000 g for 30 minutes at 4 °C. Glucose oxidase method was used to determine the fasting serum GLU, and diagnostic kits of Determiner-L TC II, Determiner-L TG, Determiner-L HDL-C and Determiner-L LDL-C (Kyowa Medex, Japan) were used to measure TC, TG, HDL and LDL on an AU5421 Automatic Biochemistry Analyzer platform (Backman-kelt, USA). AI were calculated as AI = (TC − HDL)/HDL.

### 16S rRNA gene sequencing and OTU picking

DNA of the cecum lumen samples was extracted using QIAamp DNA Stool Mini Kit (Qiagen, Germany) according to the manufacture’s standard protocol. The concentration and quality of DNA samples were measured by Nanodrop-1000 and 0.8% agrose gel. The fusion primers with dual index and adapters, including the forward primer 515F [GTGCCAGCMGCCGCGGTAA] and the reverse primer 806R [GGACTACHVGGGTWTCTAAT] were designed for amplification of the hypervariable V4 region of 16S rRNA gene under the melting temperature of 56 °C with 30 cycles. The libraries were constructed and sequenced on Illumina Miseq platform (Illumina, USA) according to the manufacturer’s protocols. The barcode and low quality sequences were removed to produce clean sequence reads. The 250-bp paired-end clean sequences were merged into tags using FLASH v.1.2.11^41^. We rarefied the library size to 10,000 tags per sample by randomly selecting the tags. Then, the 16S rRNA gene sequences were clustered into OTUs at a sequence identity threshold of 97% using the QIIME^42^. Matching OTUs to bacteria was done using a primer specific version of the GreenGenes reference database (V.13.5)^43^.

### Data analysis

We compared the alpha-diversity of cecum microbiota and the levels of serum glucose and lipids between castrated boars and gilt using the two-tailed *t-*test. Alpha-diversity was analyzed using Mothur^44^. Because some OTUs were not presented in many samples, we chose those OTUs which had ≥0.05% of relative abundance and were presented in at least 20% samples for further analysis. The association between the level of serum glucose and lipids, and the abundance of OTUs or taxonomies was analyzed using the two-part model as described by Fu *et al*.^10^. Briefly, the first part of the mode describes binomial analysis that tests for association of detecting a microbe with a trait. The binary feature of an OTU or taxonomy under study was coded as 0 for undetected or 1 for detected in each sample. The second part of quantitative analysis tests for association between trait values and the abundances of microbes, but only the samples where that microbe is present were included in analysis. *P* values obtained from the binary and quantitative analysis were further combined to calculate a meta-*P* value using an unweighted Z-score method. The minimum of the *P* values from the binary analysis, quantitative analysis and meta-analysis was set as a final association *P* value per microbe-trait pair. To correct the false discovery rate of multiple tests, 1,000× permutations were performed to determine the significance threshold. The statistical cutoff of FDR < 0.05 was set as significance threshold. This model accounts for the feature of the relative abundance of the gut microbiota. If the final *P* value came from the binary model, indicating the effect is only because of the presence/absence of the microbe, the abundance of the microbe in the tested samples is irrelevant to phenotype. If the final *P* value came from the quantitative model, this showed the abundance level of the microbe was associated with the trait. If the final *P* value came from the meta-analysis, this indicated that both the presence/absence and the abundance of the microbe had significant effect on the trait.

To get insight into the relationship between potential function capacity of gut microbiome and serum glucose and lipids, potential function capacities were predicted with the online PICRUSt software using the 16S rRNA gene sequencing data^45^. The KEGG categories and pathways that presented in >80% samples and had relative abundance>1 × 10^−4^ were used for further analysis. A univariant linear regression model was applied to evaluate the association between the concentration of serum glucose and lipids, and the relative abundance of predicted function terms in R software^46^. The association analysis was only focused on those function terms related to bacterial physiology and metabolisms. The unclassified terms were filtered from analysis. 1,000× permutations were performed to determine the significance threshold as described above. The heatmap of the KEGG terms associated with serum glucose and lipids was constructed by R software^46^.

To estimate the contribution of gut microbiome to the variation of serum glucose and lipids, a 100× cross-validation was performed as described by Fu *et al*.^10^. For each analysis, the data set was randomly split into a 70% discovery set and a 30% validation set. The risk of the gut microbiome on serum glucose and lipids (*r*_*m*_) for each animal in the validation set was estimated using an additive model: ${r}_{m}={\sum }_{j=1}^{n}({\beta }_{1}+{b}_{j}+{\beta }_{2}{q}_{j})$, where *r*_*m*_ is the serum glucose or lipid level. *n* is the number of significantly associated OTUs identified at a certain *P* value. *β*_1_ ${{\rm{\beta }}}_{1}$ and *β*_2_ ${{\rm{\beta }}}_{2}$ is the estimated effect in the binary and quantitative model, respectively. *b*_*j*_ and *q*_*j*_ represent the binary and abundance feature of *j* OTU. The range of *j* was from 1 to *n*. The explained variation was calculated as the average value of the squared correlation coefficient (*R* ^*2*^), which was corrected for the effects of sex and batch in the 100× regression analyses.

## Electronic supplementary material

Supplementary Information

## References

[1] Ramos E (2011). Replication of genome-wide association studies (GWAS) loci for fasting plasma glucose in African-Americans. Diabetologia.

[2] O’Brien RM (2013). Moving on from GWAS: functional studies on the G6PC2 gene implicated in the regulation of fasting blood glucose. Current diabetes reports.

[3] Yang H (2015). Genome-Wide Association Analysis for Blood Lipid Traits Measured in Three Pig Populations Reveals a Substantial Level of Genetic Heterogeneity. PloS one.

[4] Chasman DI (2009). Forty-three loci associated with plasma lipoprotein size, concentration, and cholesterol content in genome-wide analysis. PLoS Genet.

[5] Willer CJ, Mohlke KL (2012). Finding genes and variants for lipid levels after genome-wide association analysis. Current opinion in lipidology.

[6] Hooper LV, Macpherson AJ (2010). Immune adaptations that maintain homeostasis with the intestinal microbiota. Nature Reviews Immunology.

[7] Human Microbiome Project, C. Structure, function and diversity of the healthy human microbiome. *Nature* **486**, 207–14 (2012). 10.1038/nature11234 PMC3564958 22699609

[8] Round JL, Mazmanian SK (2009). The gut microbiota shapes intestinal immune responses during health and disease. Nat Rev Immunol.

[9] Sepp E, Kolk H, Loivukene K, Mikelsaar M (2014). Higher blood glucose level associated with body mass index and gut microbiota in elderly people. Microb Ecol Health Dis.

[10] Fu J (2015). The Gut Microbiome Contributes to a Substantial Proportion of the Variation in Blood Lipids. Circ Res.

[11] Todesco T, Rao AV, Bosello O, Jenkins DJ (1991). Propionate lowers blood glucose and alters lipid metabolism in healthy subjects. Am J Clin Nutr.

[12] Kronfeld DS (1956). Effect of butyrate administration on blood glucose in sheep. Nature.

[13] Utzschneider KM, Kratz M, Damman CJ, Hullarg M (2016). Mechanisms Linking the Gut Microbiome and Glucose Metabolism. J Clin Endocrinol Metab.

[14] Lunney JK (2007). Advances in Swine Biomedical Model Genomics. Int J Biol Sci.

[15] Xiao L (2016). A reference gene catalogue of the pig gut microbiome. Nat Microbiol.

[16] Looft T (2014). Bacteria, phages and pigs: the effects of in-feed antibiotics on the microbiome at different gut locations. ISME J.

[17] Meehan CJ, Beiko RG (2014). A phylogenomic view of ecological specialization in the Lachnospiraceae, a family of digestive tract-associated bacteria. Genome Biol Evol.

[18] Machiels K (2014). A decrease of the butyrate-producing species Roseburia hominis and Faecalibacterium prausnitzii defines dysbiosis in patients with ulcerative colitis. Gut.

[19] Khan MT (2012). The gut anaerobe Faecalibacterium prausnitzii uses an extracellular electron shuttle to grow at oxic-anoxic interphases. ISME J.

[20] Matta-el-Ammouri G, Janati-Idrissi R, Junelles AM, Petitdemange H, Gay R (1987). Effects of butyric and acetic acids on acetone-butanol formation by Clostridium acetobutylicum. Biochimie.

[21] Lecomte V (2015). Changes in gut microbiota in rats fed a high fat diet correlate with obesity-associated metabolic parameters. PLoS One.

[22] Koren O (2011). Human oral, gut, and plaque microbiota in patients with atherosclerosis. Proc Natl Acad Sci USA.

[23] Oliver MF (1995). Antioxidant nutrients, atherosclerosis, and coronary heart disease. Br Heart J.

[24] Eckburg PB (2005). Diversity of the human intestinal microbial flora. Science.

[25] Gorka P (2011). Effect of method of delivery of sodium butyrate on rumen development in newborn calves. J Dairy Sci.

[26] Anand RS, Black AL (1970). Species difference in the glucogenic behavior of butyrate in lactating ruminants. Comp Biochem Physiol.

[27] Mazzoni M (2008). Supplemental sodium butyrate stimulates different gastric cells in weaned pigs. J Nutr.

[28] Tonel I (2010). Effect of butyrate on gut development and intestinal mucosa morphology of piglets. Livestock Science.

[29] Pedersen HK (2016). Human gut microbes impact host serum metabolome and insulin sensitivity. Nature.

[30] Claus SP (2011). Colonization-induced host-gut microbial metabolic interaction. MBio.

[31] Bajaj JS (2011). Linkage of gut microbiome with cognition in hepatic encephalopathy. Ajp Gastrointestinal & Liver Physiology.

[32] Loubinoux J, Bronowicki JP, Pereira IAC, Mougenel JL, Faou AE (2002). Sulfate‐reducing bacteria in human feces and their association with inflammatory bowel diseases. Fems Microbiology Ecology.

[33] Shapiro MD, Fazio S (2016). From Lipids to Inflammation: New Approaches to Reducing Atherosclerotic Risk. Circ Res.

[34] Peters MJ (2010). The interplay between inflammation, lipids and cardiovascular risk in rheumatoid arthritis: why ratios may be better. Int J Clin Pract.

[35] Yang H (2016). Uncovering the composition of microbial community structure and metagenomics among three gut locations in pigs with distinct fatness. Sci Rep.

[36] Hu X, Wang T, Li W, Jin F, Wang L (2013). Effects of NS Lactobacillus strains on lipid metabolism of rats fed a high-cholesterol diet. Lipids Health Dis.

[37] Li F (2013). Microbiome remodelling leads to inhibition of intestinal farnesoid X receptor signalling and decreased obesity. Nat Commun.

[38] Ridlon JM, Kang DJ, Hylemon PB (2006). Bile salt biotransformations by human intestinal bacteria. J Lipid Res.

[39] Yogesh D, Halami P (2015). Evidence that multiple proteases of Bacillus subtilis can degrade fibrin and fibrinogen. International Food Research Journal.

[40] Nguimbi E (2014). Optimization of Growth, Fibrinolytic Enzyme Production and PCR Amplification of Encoding Fibrinolytic Enzyme Gene in Bacillus amyloliquefaciens Isolated from Ntoba mbodi at Brazzaville. IJSR.

[41] Magoc T, Salzberg SL (2011). FLASH: fast length adjustment of short reads to improve genome assemblies. Bioinformatics.

[42] Lawley B, Tannock GW (2017). Analysis of 16S rRNA Gene Amplicon Sequences Using the QIIME Software Package. Methods Mol Biol.

[43] Desantis TZ (2006). Greengenes: Chimera-checked 16S rRNA gene database and workbenchcompatible in ARB. Applied & Environmental Microbiology.

[44] Schloss PD (2009). Introducing mothur: open-source, platform-independent, community-supported software for describing and comparing microbial communities. Appl Environ Microbiol.

[45] Langille MGI (2013). Predictive functional profiling of microbial communities using 16S rRNA marker gene sequences. Nature Biotechnology.

[46] R Core Team R: A language and environment for statistical computing. R Foundation for Statistical Computing, Vienna, Austria. URL http://www.R-project.org/eam (2013).

