# Supplementary material for: Evaluating the contribution of gut microbiome to the variance of porcine serum glucose and lipid concentration
Source: Sci Rep. 2017 Nov 2;7:14928. doi: 10.1038/s41598-017-15044-x (PMC5668236; doi:10.1038/s41598-017-15044-x)
Supplement: Supplementary file 1 — Supplementary Information [file 41598_2017_15044_MOESM1_ESM.pdf]

Evaluating the contribution of gut microbiome to the variance of porcine serum glucose and lipid concentration

Xiaochang Huang, Shaoming Fang, Hui Yang, Jun Gao, Maozhang He, Shanlin Ke, Yuanzhang Zhao, Congying Chen *, Lusheng Huang *

Running title: Gut microbiota associated with serum lipids

<u>Address for correspondence</u>

Congying Chen (chcy75@hotmail.com), Lusheng Huang (lushenghuang@hotmail.com)

State Key Laboratory of Pig Genetic Improvement and Production Technology Jiangxi Agricultural University

Nanchang, 330045, P. R. China

Phone: 0086 - 791 - 89813080

Fax: 0086 -791 – 83900189

Supplementary Figure 1: α-diversity and microbial composition of porcine cecum luminal samples. **(a).** Boxplots of observed species and Shannon index between males and females; **(b).** Microbial composition of cecum luminal samples at phylum level and genus level.

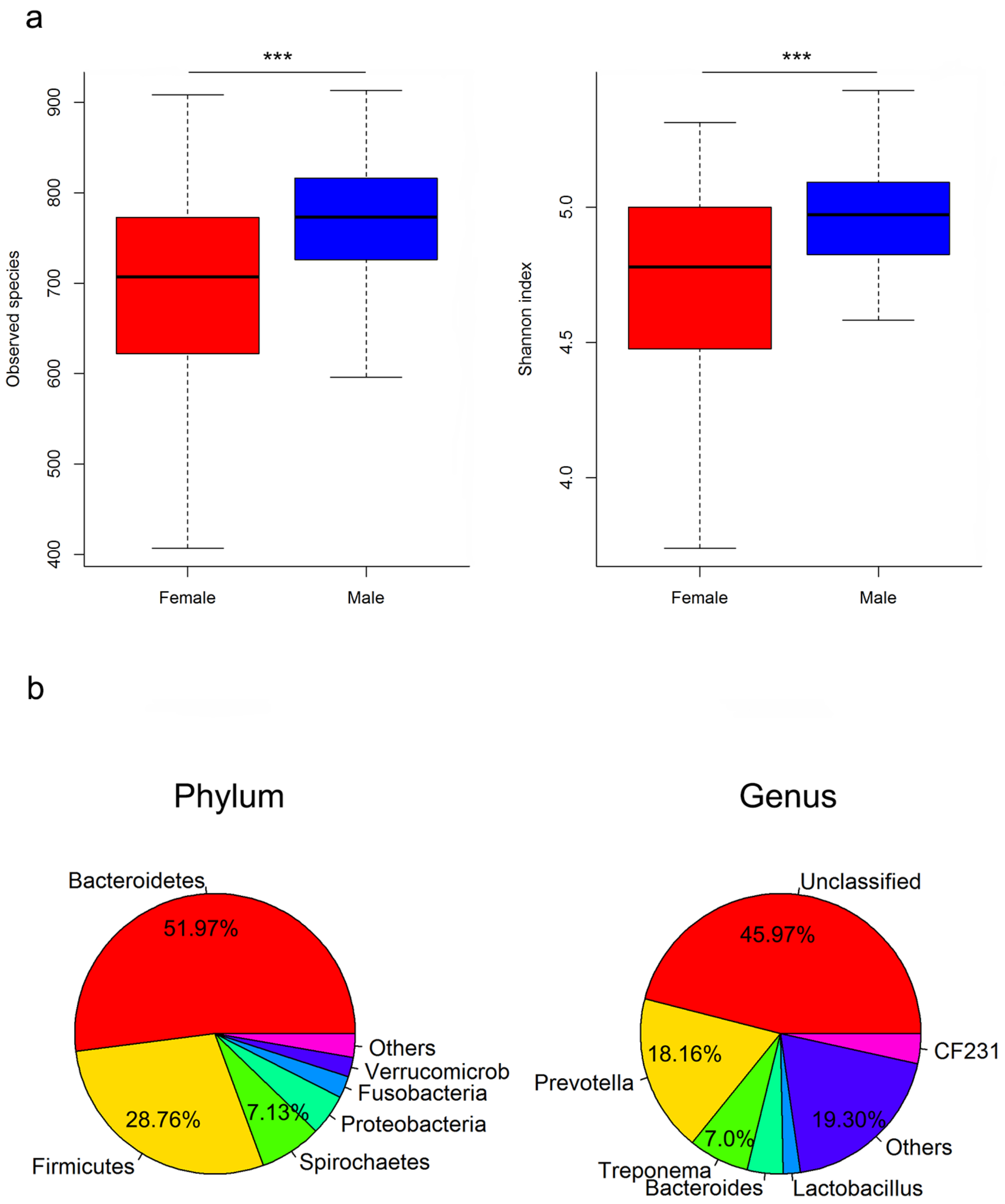

Note:

Estimate_q: The estimated regression coefficient in the quantitative model

Estimate_b: The estimated regression coefficient in the binary model

T_value_q: The test statistics T value in the quantitative model

T_value_b: The test statistics T value in the binary model

meta_z_score: the compitative unweighted Z score in the meta-analysis

P_meta: the compitative P value in the meta-analysis

final_p_value: the minimum of P values from the binary analysis, quantitative analysis and meta-analysis

Supplementary Table 1: OTUs significantly associated with blood glucose (GLU)

ID	Estimate_q*	Estimate_b	T_value_q	P_value_q	T_value_b	P_value_b	meta_z_score	P_meta	GLU_final_p_value
**Otu363_Corynebacterium**	-0.27	-0.74	-0.59	5.53E-01	-3.34	9.71E-04	-2.75	5.93E-03	9.71E-04
**Otu197_Bacteroidales**	-0.93	-1.52	-3.82	1.72E-04	-2.12	3.50E-02	-4.15	3.35E-05	3.35E-05
**Otu2316_Bacteroidales**	-0.63	-0.76	-2.02	4.57E-02	-3.37	8.74E-04	-3.77	1.66E-04	1.66E-04
**Otu1540_Bacteroidales**	1.29	0.37	2.88	4.53E-03	1.54	1.24E-01	3.09	1.98E-03	1.98E-03
**Otu1100_Bacteroidales**	-0.40	-0.66	-1.29	1.98E-01	-2.93	3.66E-03	-2.97	3.02E-03	3.02E-03
**Otu891_Bacteroides**	-0.17	-1.32	-0.55	5.82E-01	-4.69	4.61E-06	-3.63	2.85E-04	4.61E-06
**Otu160_Bacteroides**	-0.46	-1.20	-1.42	1.63E-01	-4.57	7.94E-06	-4.15	3.40E-05	7.94E-06
**Otu152_Bacteroides**	-0.38	-0.92	-1.57	1.20E-01	-4.10	5.71E-05	-3.94	7.99E-05	5.71E-05
**Otu119_Bacteroides**	0.07	-1.20	0.38	7.08E-01	-3.95	1.04E-04	2.48	1.32E-02	1.04E-04
**Otu2183_Bacteroides**	-0.11	-2.12	-0.59	5.54E-01	-3.86	1.43E-04	-3.11	1.89E-03	1.43E-04
**Otu107_Bacteroides**	-0.44	-0.72	-2.19	3.04E-02	-3.23	1.39E-03	-3.79	1.50E-04	1.50E-04
**Otu2736_Bacteroides**	-0.40	-1.34	-0.98	3.39E-01	-3.72	2.49E-04	-3.27	1.09E-03	2.49E-04
**Otu2265_Bacteroides**	-0.38	-0.84	-1.50	1.37E-01	-3.52	5.11E-04	-3.51	4.51E-04	4.51E-04
**Otu251_Bacteroides**	-0.13	-1.24	-0.24	8.15E-01	-3.43	7.01E-04	-2.56	1.04E-02	7.01E-04
**Otu2237_Bacteroides**	-0.64	-0.97	-2.54	1.18E-02	-1.88	6.10E-02	-3.11	1.90E-03	1.90E-03

**Otu32_Bacteroides_coprophilus**	-0.48	-1.18	-2.81	5.69E-03	-5.53	8.28E-08	-5.75	9.15E-09	9.15E-09
**Otu188_Bacteroides_fragilis**	-0.88	-0.71	-2.20	3.16E-02	-2.84	4.83E-03	-3.51	4.44E-04	4.44E-04
**Otu108_Bacteroides_plebeius**	-0.63	-0.64	-3.10	2.38E-03	-2.89	4.17E-03	-4.17	3.00E-05	3.00E-05
**Otu27_BS11**	-0.44	0.22	-3.10	2.25E-03	0.81	4.18E-01	1.59	1.12E-01	2.25E-03
**Otu334_Paraprevotellaceae**	-0.57	-1.01	-2.39	1.78E-02	-3.19	1.59E-03	-3.91	9.31E-05	9.31E-05
**Otu380_Paraprevotellaceae**	0.50	0.68	2.24	2.66E-02	3.02	2.79E-03	3.68	2.32E-04	2.32E-04
**Otu71_Paraprevotellaceae**	-0.32	-0.61	-1.88	6.15E-02	-2.57	1.06E-02	-3.13	1.76E-03	1.76E-03
**Otu73_CF231**	-0.72	-0.88	-3.56	4.43E-04	-1.00	3.20E-01	-3.19	1.44E-03	4.43E-04
**Otu1530_Prevotella**	0.03	-1.33	0.22	8.28E-01	-4.07	6.50E-05	2.67	7.58E-03	6.50E-05
**Otu929_Prevotella**	-0.69	-0.51	-3.22	1.51E-03	-1.56	1.20E-01	-3.34	8.31E-04	8.31E-04
**Otu47_Prevotella**	-0.37	-0.72	-1.57	1.21E-01	-2.91	3.99E-03	-3.13	1.73E-03	1.73E-03
**Otu194_Prevotella**	-0.19	-0.75	-0.80	4.22E-01	-3.17	1.73E-03	-2.78	5.40E-03	1.73E-03
**Otu51_YRC22**	-0.54	-0.20	-3.23	1.55E-03	-0.89	3.75E-01	-2.86	4.17E-03	1.55E-03
**Otu2674_Parabacteroides**	-1.42	0.11	-4.20	3.79E-05	NA	NA	-3.96	NA	3.79E-05
**Otu2269_Parabacteroides**	-1.09	-1.96	-3.25	1.31E-03	-2.24	2.59E-02	-3.85	1.19E-04	1.19E-04
**Otu255_Parabacteroides**	-1.11	-2.27	-3.52	5.16E-04	-1.30	1.96E-01	-3.37	7.51E-04	5.16E-04
**Otu960_Parabacteroides**	-0.95	-0.67	-2.67	8.10E-03	-2.12	3.49E-02	-3.36	7.68E-04	7.68E-04
**Otu688_Parabacteroides**	-0.78	-0.69	-2.52	1.24E-02	-2.13	3.44E-02	-3.26	1.10E-03	1.10E-03
**Otu2415_Parabacteroides**	-0.93	-1.09	-2.76	6.29E-03	-1.63	1.05E-01	-3.08	2.09E-03	2.09E-03
**Otu331_S24-7**	-0.51	-0.73	-1.95	5.22E-02	-2.33	2.05E-02	-3.01	2.60E-03	2.60E-03
**Otu457_YS2**	0.02	0.76	0.05	9.58E-01	3.38	8.45E-04	2.40	1.65E-02	8.45E-04
**Otu2242_YS2**	0.94	-0.01	3.15	2.01E-03	-0.04	9.70E-01	-2.16	3.10E-02	2.01E-03
**Otu23_Turicibacter**	0.81	1.27	3.75	2.20E-04	1.02	3.09E-01	3.33	8.63E-04	2.20E-04
**Otu1839_Clostridiales**	2.38	1.03	3.60	5.52E-04	4.48	1.15E-05	5.55	2.94E-08	2.94E-08
**Otu596_Clostridiales**	1.61	0.52	3.97	1.22E-04	2.33	2.04E-02	4.36	1.32E-05	1.32E-05
**Otu1638_Clostridiales**	1.23	0.69	2.76	6.64E-03	3.10	2.18E-03	4.09	4.38E-05	4.38E-05

**Otu2107_Clostridiales**	0.81	0.80	2.46	1.49E-02	3.38	8.49E-04	4.08	4.50E-05	4.50E-05
**Otu2352_Clostridiales**	1.46	0.25	3.70	3.21E-04	1.08	2.80E-01	3.31	9.37E-04	3.21E-04
**Otu697_Clostridiales**	-0.39	0.81	-0.66	5.09E-01	3.52	5.13E-04	-1.99	4.67E-02	5.13E-04
**Otu455_Clostridiales**	1.75	-0.22	3.47	7.90E-04	-0.97	3.34E-01	-1.69	9.11E-02	7.90E-04
**Otu501_Clostridiales**	1.00	0.44	2.87	4.69E-03	1.82	6.97E-02	3.28	1.03E-03	1.03E-03
**Otu1925_Clostridiales**	1.44	-0.06	3.10	2.43E-03	-0.27	7.89E-01	-1.96	5.05E-02	2.43E-03
**Otu646_Christensenellaceae**	-1.81	-0.65	-3.20	1.86E-03	-2.87	4.54E-03	-4.21	2.59E-05	2.59E-05
**Otu803_Christensenellaceae**	-0.74	-0.61	-2.04	4.29E-02	-2.14	3.31E-02	-2.94	3.30E-03	3.30E-03
**Otu231_Clostridiaceae**	0.75	1.90	3.13	2.00E-03	1.54	1.26E-01	3.27	1.08E-03	1.08E-03
**Otu33_Clostridium_butyricum**	0.64	1.28	2.65	8.64E-03	1.78	7.61E-02	3.11	1.86E-03	1.86E-03
**Otu132_Lachnospiraceae**	1.48	1.12	7.17	1.14E-11	2.71	7.25E-03	6.70	2.11E-11	1.14E-11
**Otu1914_Lachnospiraceae**	0.01	1.58	0.04	9.72E-01	5.94	1.01E-08	4.08	4.57E-05	1.01E-08
**Otu509_Lachnospiraceae**	1.11	1.17	3.90	1.34E-04	4.36	1.92E-05	5.72	1.05E-08	1.05E-08
**Otu75_Lachnospiraceae**	0.94	0.75	5.77	2.81E-08	2.14	3.36E-02	5.43	5.66E-08	2.81E-08
**Otu2502_Lachnospiraceae**	0.94	1.20	3.36	9.63E-04	4.65	5.57E-06	5.55	2.92E-08	2.92E-08
**Otu849_Lachnospiraceae**	1.11	1.27	4.04	7.45E-05	3.57	4.27E-04	5.29	1.21E-07	1.21E-07
**Otu1335_Lachnospiraceae**	1.38	0.92	3.41	8.86E-04	4.19	3.86E-05	5.26	1.43E-07	1.43E-07
**Otu196_Lachnospiraceae**	1.33	1.23	5.40	1.67E-07	1.40	1.62E-01	4.69	2.75E-06	1.67E-07
**Otu1363_Lachnospiraceae**	2.56	0.60	4.68	7.92E-06	2.70	7.50E-03	5.05	4.43E-07	4.43E-07
**Otu1639_Lachnospiraceae**	0.37	1.33	0.98	3.26E-01	5.06	8.58E-07	4.17	2.99E-05	8.58E-07
**Otu1537_Lachnospiraceae**	0.76	1.14	3.68	2.95E-04	3.27	1.25E-03	4.84	1.28E-06	1.28E-06
**Otu1168_Lachnospiraceae**	0.93	1.04	3.65	3.28E-04	3.27	1.22E-03	4.83	1.39E-06	1.39E-06
**Otu1903_Lachnospiraceae**	0.81	0.93	3.38	8.82E-04	3.40	7.96E-04	4.72	2.32E-06	2.32E-06
**Otu144_Lachnospiraceae**	0.99	0.80	4.82	2.69E-06	1.77	7.86E-02	4.56	5.06E-06	2.69E-06
**Otu225_Lachnospiraceae**	1.15	0.92	2.91	4.88E-03	3.80	1.83E-04	4.64	3.55E-06	3.55E-06
**Otu1407_Lachnospiraceae**	1.40	0.83	2.83	5.63E-03	3.73	2.35E-04	4.56	5.16E-06	5.16E-06

**Otu242_Lachnospiraceae**	1.08	0.44	4.61	7.55E-06	1.63	1.04E-01	4.32	1.59E-05	7.55E-06
**Otu1731_Lachnospiraceae**	1.43	0.86	2.47	1.53E-02	3.90	1.24E-04	4.43	9.46E-06	9.46E-06
**Otu418_Lachnospiraceae**	0.05	1.30	0.08	9.35E-01	4.51	1.02E-05	3.18	1.48E-03	1.02E-05
**Otu344_Lachnospiraceae**	0.77	1.00	2.76	6.27E-03	3.37	8.70E-04	4.29	1.81E-05	1.81E-05
**Otu1870_Lachnospiraceae**	1.05	0.81	3.34	9.97E-04	2.77	6.10E-03	4.27	1.99E-05	1.99E-05
**Otu980_Lachnospiraceae**	0.72	0.97	2.69	7.80E-03	3.42	7.43E-04	4.27	1.99E-05	1.99E-05
**Otu617_Lachnospiraceae**	1.08	0.65	4.30	2.49E-05	0.97	3.35E-01	3.66	2.50E-04	2.49E-05
**Otu442_Lachnospiraceae**	0.60	0.90	1.66	9.90E-02	4.12	5.24E-05	4.03	5.66E-05	5.24E-05
**Otu96_Lachnospiraceae**	0.92	0.85	4.06	6.68E-05	0.84	4.03E-01	3.41	6.47E-04	6.68E-05
**Otu2735_Lachnospiraceae**	1.58	0.51	3.46	7.07E-04	2.20	2.85E-02	3.94	8.04E-05	8.04E-05
**Otu770_Lachnospiraceae**	1.30	0.30	4.02	8.84E-05	1.18	2.39E-01	3.60	3.13E-04	8.84E-05
**Otu2393_Lachnospiraceae**	0.45	1.46	1.72	8.63E-02	3.84	1.60E-04	3.88	1.03E-04	1.03E-04
**Otu87_Lachnospiraceae**	1.04	0.29	3.94	1.10E-04	0.59	5.57E-01	3.15	1.64E-03	1.10E-04
**Otu559_Lachnospiraceae**	1.15	0.63	2.64	9.52E-03	2.81	5.30E-03	3.81	1.42E-04	1.42E-04
**Otu1149_Lachnospiraceae**	0.97	-0.25	3.86	1.46E-04	-0.31	7.54E-01	-2.46	1.37E-02	1.46E-04
**Otu1625_Lachnospiraceae**	0.96	1.10	3.64	3.37E-04	1.65	9.95E-02	3.70	2.16E-04	2.16E-04
**Otu2651_Lachnospiraceae**	1.06	0.57	2.72	7.60E-03	2.57	1.09E-02	3.69	2.27E-04	2.27E-04
**Otu237_Lachnospiraceae**	0.81	0.34	3.61	3.76E-04	0.43	6.64E-01	2.82	4.78E-03	3.76E-04
**Otu1756_Lachnospiraceae**	1.28	0.58	2.58	1.07E-02	2.46	1.47E-02	3.53	4.13E-04	4.13E-04
**Otu2521_Lachnospiraceae**	1.06	0.55	3.57	4.40E-04	1.41	1.59E-01	3.48	5.01E-04	4.40E-04
**Otu901_Lachnospiraceae**	0.59	0.79	1.10	2.75E-01	3.53	5.03E-04	3.23	1.23E-03	5.03E-04
**Otu244_Lachnospiraceae**	0.86	0.43	3.50	5.53E-04	0.96	3.40E-01	3.12	1.83E-03	5.53E-04
**Otu1315_Lachnospiraceae**	0.82	0.68	2.25	2.55E-02	2.66	8.24E-03	3.45	5.65E-04	5.65E-04
**Otu2206_Lachnospiraceae**	0.22	0.77	0.69	4.90E-01	3.48	5.89E-04	2.92	3.53E-03	5.89E-04
**Otu1014_Lachnospiraceae**	0.82	0.61	2.11	3.69E-02	2.74	6.59E-03	3.40	6.81E-04	6.81E-04
**Otu2170_Lachnospiraceae**	0.91	0.62	2.05	4.27E-02	2.78	5.92E-03	3.38	7.26E-04	7.26E-04

**Otu677_Lachnospiraceae**	1.23	0.81	1.58	1.19E-01	3.16	1.80E-03	3.31	9.31E-04	9.31E-04
**Otu65_Lachnospiraceae**	0.67	1.32	3.33	1.01E-03	0.75	4.54E-01	2.85	4.30E-03	1.01E-03
**Otu1498_Lachnospiraceae**	0.92	1.72	3.20	1.58E-03	1.39	1.66E-01	3.21	1.31E-03	1.31E-03
**Otu292_Lachnospiraceae**	-0.25	0.86	-0.36	7.23E-01	3.19	1.63E-03	-1.98	4.80E-02	1.63E-03
**Otu1778_Lachnospiraceae**	1.54	0.45	2.52	1.36E-02	1.97	5.02E-02	3.13	1.74E-03	1.74E-03
**Otu129_Lachnospiraceae**	0.52	0.53	2.36	1.94E-02	2.04	4.24E-02	3.09	2.01E-03	2.01E-03
**Otu1652_Lachnospiraceae**	-0.26	0.69	-0.39	6.96E-01	3.12	2.03E-03	-1.91	5.67E-02	2.03E-03
**Otu136_Lachnospiraceae**	0.89	0.11	3.12	2.03E-03	NA	NA	2.87	NA	2.03E-03
**Otu2673_Lachnospiraceae**	0.75	0.71	1.73	8.87E-02	2.68	7.89E-03	3.08	2.05E-03	2.05E-03
**Otu1398_Lachnospiraceae**	0.86	0.35	2.99	3.25E-03	1.41	1.60E-01	3.07	2.11E-03	2.11E-03
**Otu200_Lachnospiraceae**	0.83	1.29	2.86	4.67E-03	1.47	1.43E-01	3.04	2.40E-03	2.40E-03
**Otu921_Lachnospiraceae**	1.27	0.42	2.48	1.43E-02	1.85	6.57E-02	3.03	2.42E-03	2.42E-03
**Otu1340_Lachnospiraceae**	0.85	0.57	2.20	2.89E-02	2.05	4.16E-02	2.99	2.82E-03	2.82E-03
**Otu2618_Lachnospiraceae**	0.85	0.07	3.00	3.05E-03	0.28	7.79E-01	2.29	2.18E-02	3.05E-03
**Otu241_Lachnospiraceae**	0.85	0.83	1.36	1.82E-01	2.88	4.37E-03	2.96	3.09E-03	3.09E-03
**Otu713_Lachnospiraceae**	-0.01	0.69	-0.02	9.81E-01	2.98	3.23E-03	-2.07	3.89E-02	3.23E-03
**Otu449_Lachnospiraceae**	0.96	0.39	2.44	1.59E-02	1.72	8.62E-02	2.92	3.51E-03	3.51E-03
**Otu385_Anaerostipes**	1.14	0.75	1.89	6.31E-02	3.04	2.67E-03	3.44	5.87E-04	5.87E-04
**Otu652_Blautia**	1.75	0.27	4.52	1.29E-05	1.17	2.45E-01	3.91	9.35E-05	1.29E-05
**Otu1994_Blautia**	1.06	0.61	2.49	1.40E-02	2.66	8.42E-03	3.60	3.16E-04	3.16E-04
**Otu180_Coprococcus**	1.08	1.03	4.82	2.72E-06	3.09	2.27E-03	5.48	4.36E-08	4.36E-08
**Otu291_Coprococcus**	1.30	0.63	5.14	7.36E-07	2.49	1.35E-02	5.25	1.53E-07	1.53E-07
**Otu908_Coprococcus**	1.05	0.97	3.05	2.65E-03	4.23	3.38E-05	5.06	4.25E-07	4.25E-07
**Otu285_Coprococcus**	0.92	0.88	3.80	1.93E-04	3.17	1.70E-03	4.86	1.20E-06	1.20E-06
**Otu428_Coprococcus**	1.02	0.91	2.44	1.67E-02	4.11	5.50E-05	4.54	5.51E-06	5.51E-06
**Otu612_Coprococcus**	0.60	0.99	1.42	1.57E-01	4.39	1.67E-05	4.04	5.25E-05	1.67E-05

**Otu2686_Coprococcus**	0.57	0.93	1.92	5.69E-02	3.98	9.29E-05	4.11	3.96E-05	3.96E-05
**Otu469_Coprococcus**	1.47	0.53	2.77	7.06E-03	2.22	2.73E-02	3.47	5.28E-04	5.28E-04
**Otu541_Coprococcus**	0.37	0.76	1.30	1.94E-01	2.89	4.17E-03	2.94	3.24E-03	3.24E-03
**Otu217_Lachnospira**	0.94	0.92	3.70	2.97E-04	3.84	1.58E-04	5.23	1.70E-07	1.70E-07
**Otu470_Lachnospira**	1.22	0.27	4.07	7.26E-05	1.09	2.75E-01	3.58	3.47E-04	7.26E-05
**Otu352_Pseudobutyrivibrio**	0.96	0.35	3.31	1.12E-03	1.38	1.68E-01	3.28	1.05E-03	1.05E-03
**Otu111_Roseburia**	1.48	0.11	5.85	1.65E-08	NA	NA	5.52	NA	1.65E-08
**Otu2600_Roseburia**	1.52	0.11	5.17	4.85E-07	NA	NA	4.90	NA	4.85E-07
**Otu247_Roseburia**	-0.62	0.99	-0.91	3.66E-01	3.33	1.01E-03	-1.69	9.19E-02	1.01E-03
**Otu684_Mogibacteriaceae**	-1.12	-0.46	-2.54	1.22E-02	-2.06	4.05E-02	-3.22	1.28E-03	1.28E-03
**Otu872_Peptococcaceae**	-1.54	-0.12	-3.22	1.61E-03	-0.52	6.02E-01	-2.60	9.35E-03	1.61E-03
**Otu302_Peptococcus**	0.79	0.72	1.86	6.77E-02	2.87	4.49E-03	3.30	9.64E-04	9.64E-04
**Otu138_Ruminococcaceae**	-0.74	-0.77	-3.90	1.40E-04	-3.17	1.73E-03	-4.91	9.21E-07	9.21E-07
**Otu2072_Ruminococcaceae**	1.30	0.72	3.20	1.67E-03	3.21	1.53E-03	4.46	8.07E-06	8.07E-06
**Otu315_Ruminococcaceae**	-1.23	-0.34	-4.30	3.29E-05	-1.48	1.39E-01	-3.98	6.86E-05	3.29E-05
**Otu318_Ruminococcaceae**	-0.89	-0.68	-2.76	6.67E-03	-3.07	2.42E-03	-4.06	4.85E-05	4.85E-05
**Otu254_Ruminococcaceae**	-1.20	-0.56	-3.16	2.00E-03	-2.50	1.32E-02	-3.94	8.21E-05	8.21E-05
**Otu1116_Ruminococcaceae**	-1.17	-0.41	-3.65	3.72E-04	-1.82	6.94E-02	-3.80	1.44E-04	1.44E-04
**Otu811_Ruminococcaceae**	1.51	0.62	2.29	2.36E-02	2.77	6.03E-03	3.54	3.97E-04	3.97E-04
**Otu89_Ruminococcaceae**	-0.67	-0.12	-3.38	8.51E-04	-0.28	7.82E-01	-2.55	1.06E-02	8.51E-04
**Otu952_Ruminococcaceae**	1.13	0.55	2.20	2.90E-02	2.40	1.73E-02	3.23	1.25E-03	1.25E-03
**Otu92_Ruminococcaceae**	-0.57	-0.38	-2.98	3.26E-03	-1.51	1.32E-01	-3.14	1.67E-03	1.67E-03
**Otu491_Ruminococcaceae**	-0.64	-0.53	-2.14	3.41E-02	-2.09	3.79E-02	-2.97	3.02E-03	3.02E-03
**Otu2095_Faecalibacterium**	1.51	0.69	2.57	1.45E-02	2.21	2.77E-02	3.29	1.02E-03	1.02E-03
**Otu266_Faecalibacterium**	1.17	0.34	4.36	2.05E-05	1.13	2.59E-01	3.81	1.39E-04	2.05E-05
**Otu240_Faecalibacterium**	0.97	0.35	2.84	5.37E-03	1.56	1.21E-01	3.07	2.17E-03	2.17E-03

**Otu1484_Oscillospira**	-1.01	-0.26	-3.80	1.85E-04	-0.50	6.15E-01	-3.00	2.71E-03	1.85E-04
**Otu833_Oscillospira**	1.04	0.49	3.12	2.08E-03	1.52	1.31E-01	3.25	1.17E-03	1.17E-03
**Otu487_Oscillospira**	-0.91	-0.51	-1.95	5.41E-02	-2.23	2.65E-02	-2.93	3.38E-03	3.38E-03
**Otu427_Ruminococcus**	1.07	0.65	3.23	1.53E-03	2.77	6.01E-03	4.18	2.88E-05	2.88E-05
**Otu1952_Ruminococcus**	1.38	0.68	2.81	5.97E-03	3.01	2.87E-03	4.05	5.07E-05	5.07E-05
**Otu521_Ruminococcus**	0.79	0.84	3.27	1.25E-03	2.12	3.52E-02	3.77	1.62E-04	1.62E-04
**Otu391_Ruminococcus**	0.53	1.27	2.03	4.35E-02	3.31	1.08E-03	3.74	1.84E-04	1.84E-04
**Otu328_Ruminococcus**	0.56	0.65	2.25	2.57E-02	2.61	9.76E-03	3.41	6.62E-04	6.62E-04
**Otu337_Ruminococcus**	0.71	0.70	1.69	9.33E-02	3.14	1.92E-03	3.38	7.24E-04	7.24E-04
**Otu1616_Ruminococcus**	0.76	0.45	3.04	2.69E-03	1.19	2.37E-01	2.96	3.09E-03	2.69E-03
**Otu379_Ruminococcus**	0.65	0.49	2.44	1.56E-02	1.76	7.96E-02	2.95	3.18E-03	3.18E-03
**Otu97_Anaerovibrio**	0.65	0.65	2.09	3.92E-02	2.84	4.84E-03	3.45	5.60E-04	5.60E-04
**Otu181_Megasphaera**	0.49	-0.98	1.33	1.94E-01	-3.12	2.06E-03	1.26	2.07E-01	2.06E-03
**Otu759_p-75-a5**	1.61	0.25	3.36	1.04E-03	1.11	2.70E-01	3.10	1.94E-03	1.04E-03
**Otu862_Fusobacteriaceae**	-0.66	-1.01	-3.51	6.53E-04	-4.62	6.31E-06	-5.60	2.10E-08	2.10E-08
**Otu3_Fusobacteriaceae**	-0.59	-0.45	-5.06	9.41E-07	-1.44	1.50E-01	-4.49	7.29E-06	9.41E-07
**Otu840_Fusobacteriaceae**	-0.58	-0.68	-2.91	4.51E-03	-3.01	2.93E-03	-4.11	3.93E-05	3.93E-05
**Otu2231_Fusobacteriaceae**	-0.68	-1.17	-1.03	3.14E-01	-3.35	9.34E-04	-3.05	2.27E-03	9.34E-04
**Otu43_Fusobacterium**	-0.38	-0.82	-1.47	1.46E-01	-3.12	2.04E-03	-3.21	1.34E-03	1.34E-03
**Otu28_Fusobacterium**	-0.34	-0.54	-2.47	1.44E-02	-1.96	5.17E-02	-3.11	1.90E-03	1.90E-03
**Otu319_Proteobacteria**	-0.27	-0.96	-1.06	2.90E-01	-4.21	3.66E-05	-3.67	2.45E-04	3.66E-05
**Otu321_Betaproteobacteria**	-0.19	-0.83	-0.83	4.08E-01	-3.18	1.66E-03	-2.81	4.98E-03	1.66E-03
**Otu112_Desulfovibrionaceae**	-0.67	-0.81	-3.20	1.62E-03	-3.31	1.09E-03	-4.54	5.69E-06	5.69E-06
**Otu591_Desulfovibrionaceae**	-1.12	-0.19	-4.33	2.42E-05	-0.61	5.42E-01	-3.42	6.33E-04	2.42E-05
**Otu199_Enterobacteriaceae**	-0.10	-0.69	-0.36	7.20E-01	-2.98	3.22E-03	-2.34	1.94E-02	3.22E-03
**Otu2813_Treponema**	0.82	0.51	2.30	2.34E-02	2.30	2.23E-02	3.22	1.28E-03	1.28E-03

**Otu1005_Treponema**	0.70	0.44	2.72	7.22E-03	1.71	8.95E-02	3.10	1.93E-03	1.93E-03
**Otu437_Anaeroplasma**	0.46	0.91	0.70	4.84E-01	3.65	3.23E-04	3.04	2.38E-03	3.23E-04
**Otu527_RF39**	1.24	0.77	2.63	9.77E-03	3.50	5.54E-04	4.27	1.97E-05	1.97E-05
**Otu484_RF39**	1.79	0.50	3.07	3.00E-03	2.07	3.93E-02	3.56	3.77E-04	3.77E-04
**Otu1146_RF39**	1.63	0.60	2.34	2.13E-02	2.71	7.27E-03	3.53	4.22E-04	4.22E-04
**Otu249_RF39**	0.68	0.51	2.50	1.40E-02	2.29	2.26E-02	3.35	8.11E-04	8.11E-04
**Otu278_RFP12**	-0.52	-0.80	-2.61	9.57E-03	-2.03	4.38E-02	-3.26	1.12E-03	1.12E-03
**Otu311_RFP12**	-0.62	-0.34	-2.99	3.21E-03	-1.30	1.94E-01	-3.00	2.68E-03	2.68E-03

Supplementary table 2: OTUs significantly associated with total cholesterol (TC)

**ID**	**Estimate_q**	**Estimate_b**	**T_value_q**	**P_value_q**	**T_value_b**	**P_value_b**	**meta_z_score**	**P_meta**	**final_p_value**
**Otu307_Treponema**	0.02	-0.27	0.41	6.81E-01	-5.4	1.51E-07	3.42	6.20E-04	1.51E-07
**Otu1069_Treponema**	-0.03	-0.18	-0.53	5.95E-01	-4.0	8.02E-05	-3.16	1.55E-03	8.02E-05
**Otu169_Prevotella**	-0.02	-0.19	-0.45	6.55E-01	-4.0	8.72E-05	-3.09	2.00E-03	8.72E-05
**Otu2043_Ruminococcaceae**	-0.09	-0.18	-0.77	4.43E-01	-4.0	9.17E-05	-3.31	9.40E-04	9.17E-05
**Otu68_Prevotella_stercorea**	0.02	0.19	0.76	4.48E-01	3.9	1.46E-04	3.22	1.27E-03	1.46E-04
**Otu1047_Treponema**	0.02	-0.24	0.64	5.23E-01	-3.7	2.51E-04	2.14	3.26E-02	2.51E-04
**Otu2306_Ruminococcaceae**	-0.23	-0.13	-4.77	4.04E-06	-2.8	5.37E-03	-5.23	1.71E-07	1.71E-07
**Otu13_Lactobacillus**	-0.09	-0.22	-3.44	6.83E-04	-2.5	1.33E-02	-4.15	3.29E-05	3.29E-05
**Otu521_Ruminococcus**	-0.12	-0.25	-2.58	1.06E-02	-3.3	1.32E-03	-4.08	4.55E-05	4.55E-05
**Otu2206_Lachnospiraceae**	0.12	0.15	2.21	2.90E-02	3.6	3.91E-04	4.05	5.09E-05	5.09E-05
**Otu1457_Ruminococcus**	-0.20	-0.14	-2.43	1.66E-02	-3.3	9.57E-04	-4.03	5.58E-05	5.58E-05
**Otu456_Ruminococcaceae**	0.17	0.14	2.63	9.42E-03	3.0	3.30E-03	3.91	9.08E-05	9.08E-05
**Otu350_Ruminococcus**	-0.14	-0.13	-2.90	4.18E-03	-2.5	1.22E-02	-3.80	1.46E-04	1.46E-04
**Otu117_Ruminococcaceae**	-0.18	0.03	-4.20	3.77E-05	0.2	8.78E-01	2.81	5.03E-03	3.77E-05
**Otu127_GMD14H09**	0.14	0.04	3.70	2.85E-04	0.8	4.21E-01	3.13	1.72E-03	2.85E-04

Supplementary table 3: OTUs significantly associated with ratio of low density lipoprotein to high density lipoprotein (LDL/HDL)

ID	Estimate_q	Estimate_b	T_value_q	P_value_q	T_value_b	P_value_b	meta_z_score	P_meta	final_p_value
**Otu30_Bacteroidetes**	-0.11	-1.14	-2.57	1.08E-02	-5.26	3.17E-07	-5.42	6.05E-08	6.05E-08
**Otu862_Fusobacteriaceae**	0.25	0.34	3.09	2.52E-03	4.60	6.97E-06	5.31	1.07E-07	1.07E-07
**Otu199_Enterobacteriaceae**	0.36	0.22	4.01	1.31E-04	2.85	4.74E-03	4.70	2.58E-06	2.58E-06
**Otu3_Fusobacteriaceae**	0.19	0.16	4.75	3.94E-06	1.47	1.44E-01	4.30	1.73E-05	3.94E-06
**Otu328_Ruminococcus**	-0.02	-0.39	-0.24	8.14E-01	-4.63	6.14E-06	-3.36	7.69E-04	6.14E-06
**Otu33_Clostridium_butyricum**	-0.36	-0.29	-4.51	1.05E-05	-1.16	2.46E-01	-3.94	8.27E-05	1.05E-05
**Otu43_Fusobacterium**	0.28	0.35	2.36	2.22E-02	3.95	1.05E-04	4.36	1.30E-05	1.30E-05
**Otu231_Clostridiaceae**	-0.36	-0.22	-4.42	1.53E-05	-0.51	6.11E-01	-3.42	6.32E-04	1.53E-05
**Otu394_Epulopiscium**	-0.07	-0.41	-0.82	4.11E-01	-4.38	1.78E-05	-3.62	2.99E-04	1.78E-05
**Otu2813_Treponema**	-0.25	-0.28	-2.36	1.99E-02	-3.75	2.25E-04	-4.26	2.09E-05	2.09E-05
**Otu2402_Treponema**	-0.38	-0.17	-3.89	1.53E-04	-2.21	2.79E-02	-4.23	2.32E-05	2.32E-05
**Otu259_Oscillospira**	0.29	0.27	3.09	2.31E-03	2.80	5.51E-03	4.12	3.83E-05	3.83E-05
**Otu15_Clostridium**	-0.36	0.04	-4.13	4.94E-05	NA	NA	-3.89	NA	4.94E-05
**Otu1922_Treponema**	-0.26	-0.27	-2.22	2.83E-02	-3.59	4.08E-04	-4.05	5.12E-05	5.12E-05
**Otu151_Prevotella**	-0.05	-0.39	-0.73	4.69E-01	-4.06	6.73E-05	-3.33	8.67E-04	6.73E-05
**Otu1384_Ruminococcaceae**	0.51	0.14	3.93	1.28E-04	1.78	7.66E-02	3.96	7.48E-05	7.48E-05
**Otu47_Prevotella**	-0.05	0.33	-0.45	6.51E-01	3.99	8.75E-05	-2.45	1.41E-02	8.75E-05
**Otu1014_Lachnospiraceae**	0.11	-0.30	1.15	2.51E-01	-3.99	8.85E-05	1.96	5.00E-02	8.85E-05
**Otu45_Treponema**	-0.20	-0.14	-3.99	8.88E-05	-1.04	3.00E-01	-3.50	4.58E-04	8.88E-05
**Otu101_Epulopiscium**	0.31	0.26	2.33	2.27E-02	3.30	1.13E-03	3.91	9.10E-05	9.10E-05
**Otu1410_Treponema**	-0.34	-0.15	-3.84	1.73E-04	-1.76	7.90E-02	-3.90	9.73E-05	9.73E-05
**Otu193_Mogibacteriaceae**	0.32	0.64	3.88	1.34E-04	1.52	1.30E-01	3.77	1.63E-04	1.34E-04
**Otu5_Escherichia**	0.16	0.27	3.87	1.42E-04	1.33	1.86E-01	3.63	2.88E-04	1.42E-04
**Otu1975_Treponema**	-0.25	-0.22	-2.63	9.45E-03	-2.79	5.73E-03	-3.79	1.51E-04	1.51E-04

**Otu366_Anaeroplasma**	-0.32	-0.19	-2.99	3.44E-03	-2.43	1.56E-02	-3.78	1.58E-04	1.58E-04
**Otu17_Phascolarctobacterium**	0.36	0.04	3.80	1.87E-04	NA	NA	3.56	NA	1.87E-04
**Otu13_Lactobacillus**	-0.18	-0.10	-3.78	2.02E-04	-0.62	5.33E-01	-3.07	2.15E-03	2.02E-04
**Otu16_Treponema**	-0.13	-0.42	-2.79	5.77E-03	-2.47	1.41E-02	-3.69	2.26E-04	2.26E-04
**Otu1047_Treponema**	-0.11	-0.38	-1.92	5.62E-02	-3.34	9.84E-04	-3.68	2.33E-04	2.33E-04
**Otu2022_Ruminococcus**	0.16	-0.29	1.20	2.34E-01	-3.73	2.40E-04	1.76	7.91E-02	2.40E-04
**Otu188_Bacteroides_fragilis**	0.10	0.31	0.65	5.21E-01	3.68	2.85E-04	3.02	2.53E-03	2.85E-04
**Otu649_Mogibacteriaceae**	0.15	0.28	0.78	4.35E-01	3.68	2.86E-04	3.12	1.83E-03	2.86E-04
**Otu1151_Prevotella**	-0.17	-0.46	-2.17	3.10E-02	-3.00	2.99E-03	-3.62	2.89E-04	2.89E-04
**Otu485_Epulopiscium**	-0.04	-0.30	-0.37	7.13E-01	-3.65	3.20E-04	-2.80	5.03E-03	3.20E-04
**Otu1616_Ruminococcus**	-0.29	-0.12	-3.66	3.20E-04	-0.94	3.46E-01	-3.21	1.32E-03	3.20E-04
**Otu42_Actinobacillus**	0.19	0.19	2.66	8.89E-03	2.48	1.38E-02	3.59	3.29E-04	3.29E-04
**Otu186_Prevotella**	-0.16	-0.38	-2.38	1.84E-02	-2.74	6.59E-03	-3.59	3.32E-04	3.32E-04
**Otu2674_Parabacteroides**	0.42	0.04	3.63	3.48E-04	NA	NA	3.39	NA	3.48E-04
**Otu2502_Lachnospiraceae**	-0.16	-0.30	-1.71	8.89E-02	-3.40	8.04E-04	-3.57	3.53E-04	3.53E-04
**Otu442_Lachnospiraceae**	-0.01	-0.27	-0.14	8.91E-01	-3.60	3.82E-04	-2.61	9.09E-03	3.82E-04
**Otu1800_Parabacteroides**	0.39	0.20	3.60	3.87E-04	0.88	3.81E-01	3.13	1.76E-03	3.87E-04
**Otu135_CF231**	0.28	0.24	2.42	1.86E-02	2.68	7.94E-03	3.54	3.98E-04	3.98E-04
**Otu111_Roseburia**	-0.32	0.04	-3.59	3.99E-04	NA	NA	-3.35	NA	3.99E-04
**Otu291_Coprococcus**	0.00	-0.30	0.01	9.91E-01	-3.58	4.15E-04	2.49	1.28E-02	4.15E-04
**Otu31_Treponema**	-0.24	-0.30	-3.57	4.28E-04	-1.00	3.19E-01	-3.19	1.40E-03	4.28E-04
**Otu104_Lactobacillus_reuteri**	-0.11	-0.52	-2.03	4.39E-02	-2.98	3.14E-03	-3.51	4.42E-04	4.42E-04
**Otu2449_Lachnospiraceae**	-0.05	-0.39	-0.54	5.88E-01	-3.56	4.47E-04	-2.87	4.16E-03	4.47E-04
**Otu2562_Mogibacteriaceae**	0.32	0.22	3.36	9.08E-04	1.65	1.00E-01	3.51	4.52E-04	4.52E-04
**Otu350_Ruminococcus**	-0.23	-0.22	-2.47	1.45E-02	-2.52	1.25E-02	-3.49	4.76E-04	4.76E-04
**Otu2306_Ruminococcaceae**	-0.09	-0.29	-1.03	3.04E-01	-3.53	4.98E-04	-3.19	1.43E-03	4.98E-04

**Otu468_Ruminococcus**	-0.04	-0.26	-0.43	6.65E-01	-3.50	5.57E-04	-2.75	6.01E-03	5.57E-04
**Otu2768_Clostridiaceae**	-0.12	-0.27	-1.03	3.04E-01	-3.50	5.61E-04	-3.17	1.54E-03	5.61E-04
**Otu816_Peptococcaceae**	0.03	0.26	0.16	8.69E-01	3.47	6.11E-04	2.54	1.11E-02	6.11E-04
**Otu32_Bacteroides_coprophilus**	0.11	0.26	1.47	1.45E-01	3.43	7.16E-04	3.42	6.17E-04	6.17E-04
**Otu141_S24-7**	0.20	-0.23	3.44	6.96E-04	-0.66	5.08E-01	-1.93	5.36E-02	6.96E-04
**Otu2231_Fusobacteriaceae**	0.50	0.38	1.66	1.10E-01	3.21	1.49E-03	3.38	7.36E-04	7.36E-04
**Otu44_Prevotella**	-0.14	-0.38	-2.56	1.11E-02	-2.25	2.55E-02	-3.37	7.39E-04	7.39E-04
**Otu1423_Treponema**	-0.42	-0.13	-3.33	1.07E-03	-1.50	1.35E-01	-3.37	7.51E-04	7.51E-04
**Otu195_Pasteurellaceae**	0.28	0.21	2.22	2.95E-02	2.60	9.82E-03	3.37	7.65E-04	7.65E-04
**Otu575_Ruminococcaceae**	0.00	-0.26	-0.01	9.93E-01	-3.41	7.69E-04	-2.38	1.71E-02	7.69E-04
**Otu872_Peptococcaceae**	0.27	0.24	1.60	1.11E-01	3.19	1.59E-03	3.36	7.80E-04	7.80E-04
**Otu106_Ruminococcus**	-0.15	-0.25	-2.44	1.54E-02	-2.34	2.02E-02	-3.36	7.91E-04	7.91E-04
**Otu161_rc4-4**	0.29	0.32	3.38	8.36E-04	1.39	1.66E-01	3.34	8.30E-04	8.30E-04
**Otu89_Ruminococcaceae**	0.23	0.10	3.38	8.64E-04	0.68	4.96E-01	2.84	4.55E-03	8.64E-04
**Otu413_Lachnospiraceae**	0.06	-0.25	0.47	6.43E-01	-3.35	9.36E-04	2.01	4.42E-02	9.36E-04
**Otu756_Dethiosulfovibrionaceae**	0.28	0.23	2.03	4.40E-02	2.69	7.69E-03	3.31	9.38E-04	9.38E-04
**Otu243_Lachnospiraceae**	-0.29	0.01	-3.36	9.72E-04	0.16	8.71E-01	2.22	2.66E-02	9.72E-04
**Otu2633_Phascolarctobacterium**	0.29	0.01	3.31	1.09E-03	0.11	9.10E-01	2.39	1.69E-02	1.09E-03
**Otu276_Ruminococcaceae**	0.28	0.22	1.81	7.35E-02	2.84	4.89E-03	3.26	1.13E-03	1.13E-03
**Otu683_Ruminococcaceae**	-0.36	-0.18	-2.31	2.29E-02	-2.34	2.00E-02	-3.25	1.14E-03	1.14E-03
**Otu432_Ruminococcus**	-0.32	0.33	-1.33	1.92E-01	3.29	1.14E-03	-1.38	1.69E-01	1.14E-03
**Otu1069_Treponema**	-0.27	-0.19	-2.34	2.04E-02	-2.28	2.34E-02	-3.24	1.18E-03	1.18E-03
**Otu596_Clostridiales**	-0.02	-0.25	-0.20	8.45E-01	-3.25	1.33E-03	-2.41	1.61E-02	1.33E-03

Supplementary table 4: OTUs significantly associated with atherosclerosis index (AI)

ID	Estimate_q	Estimate_b	T_value_q	P_value_q	T_value_b	P_value_b	meta_z_score	P_meta	final_p_value
**Otu862_Fusobacteriaceae**	0.31	0.40	3.09	2.53E-03	4.22	3.5E-05	5.06	4.11E-07	4.11E-07
**Otu13_Lactobacillus**	-0.30	-0.32	-5.08	7.96E-07	-1.58	1.1E-01	-4.61	4.12E-06	7.96E-07
**Otu30_Bacteroidetes**	-0.17	-1.08	-3.00	3.00E-03	-3.78	2.0E-04	-4.73	2.22E-06	2.22E-06
**Otu104_Lactobacillus_reuteri**	-0.24	-0.71	-3.42	7.32E-04	-3.20	1.6E-03	-4.62	3.79E-06	3.79E-06
**Otu350_Ruminococcus**	-0.34	-0.39	-3.03	2.79E-03	-3.43	7.0E-04	-4.51	6.46E-06	6.46E-06
**Otu328_Ruminococcus**	0.01	-0.49	0.14	8.86E-01	-4.61	6.7E-06	3.08	2.05E-03	6.69E-06
**Otu15_Clostridium**	-0.51	0.05	-4.58	7.55E-06	NA	NA	-4.33	NA	7.55E-06
**Otu2633_Phascolarctobacterium**	0.50	0.05	4.59	7.74E-06	0.30	7.7E-01	3.37	7.44E-04	7.74E-06
**Otu394_Epulopiscium**	-0.15	-0.55	-1.33	1.85E-01	-4.52	9.7E-06	-4.07	4.76E-05	9.66E-06
**Otu231_Clostridiaceae**	-0.46	-0.11	-4.48	1.16E-05	-0.20	8.4E-01	-3.24	1.19E-03	1.16E-05
**Otu17_Phascolarctobacterium**	0.54	0.05	4.48	1.18E-05	NA	NA	4.23	NA	1.18E-05
**Otu106_Ruminococcus**	-0.26	-0.39	-3.22	1.51E-03	-2.84	4.9E-03	-4.23	2.29E-05	2.29E-05
**Otu3_Fusobacteriaceae**	0.22	0.19	4.32	2.40E-05	1.42	1.6E-01	3.99	6.66E-05	2.40E-05
**Otu199_Enterobacteriaceae**	0.39	0.26	3.49	7.54E-04	2.61	9.8E-03	4.21	2.56E-05	2.56E-05
**Otu135_CF231**	0.40	0.32	3.21	2.19E-03	2.88	4.4E-03	4.18	2.90E-05	2.90E-05
**Otu816_Peptococcaceae**	0.00	0.40	0.01	9.91E-01	4.22	3.4E-05	2.94	3.31E-03	3.44E-05
**Otu2306_Ruminococcaceae**	-0.23	-0.41	-2.05	4.17E-02	-3.84	1.6E-04	-4.11	3.96E-05	3.96E-05
**Otu756_Dethiosulfovibrionaceae**	0.41	0.38	2.38	1.84E-02	3.49	5.8E-04	4.10	4.15E-05	4.15E-05
**Otu1616_Ruminococcus**	-0.44	-0.08	-4.13	5.13E-05	-0.50	6.2E-01	-3.22	1.29E-03	5.13E-05
**Otu43_Fusobacterium**	0.31	0.39	2.39	2.06E-02	3.41	7.7E-04	4.02	5.94E-05	5.94E-05
**Otu141_S24-7**	0.31	-0.12	4.06	6.82E-05	-0.27	7.9E-01	-2.63	8.59E-03	6.82E-05
**Otu16_Treponema**	-0.16	-0.61	-2.71	7.21E-03	-2.85	4.7E-03	-3.90	9.67E-05	9.67E-05
**Otu33_Clostridium_butyricum**	-0.41	-0.34	-3.96	1.01E-04	-1.08	2.8E-01	-3.51	4.50E-04	1.01E-04
**Otu2022_Ruminococcus**	-0.06	-0.39	-0.29	7.69E-01	-3.94	1.1E-04	-2.94	3.23E-03	1.08E-04

**Otu123_**	0.32	-0.03	3.92	1.18E-04	-0.11	9.1E-01	-2.64	8.21E-03	1.18E-04
**Otu366_Anaeroplasma**	-0.44	-0.24	-3.04	2.96E-03	-2.49	1.4E-02	-3.85	1.20E-04	1.20E-04
**Otu47_Prevotella**	-0.13	0.42	-1.01	3.15E-01	3.90	1.3E-04	-2.00	4.55E-02	1.26E-04
**Otu376_Ruminococcus**	-0.32	-0.42	-2.52	1.24E-02	-2.93	3.7E-03	-3.82	1.35E-04	1.35E-04
**Otu872_Peptococcaceae**	0.17	0.37	0.79	4.31E-01	3.81	1.8E-04	3.21	1.33E-03	1.77E-04
**Otu1749_Phascolarctobacterium**	0.29	0.16	3.80	1.86E-04	1.00	3.2E-01	3.35	8.18E-04	1.86E-04
**Otu1432_Lactobacillus_reuteri**	-0.30	-0.28	-3.59	4.09E-04	-1.74	8.3E-02	-3.73	1.94E-04	1.94E-04
**Otu1047_Treponema**	-0.07	-0.54	-0.96	3.37E-01	-3.77	2.1E-04	-3.30	9.64E-04	2.09E-04
**Otu1014_Lachnospiraceae**	0.10	-0.36	0.76	4.49E-01	-3.72	2.5E-04	2.05	4.01E-02	2.53E-04
**Otu151_Prevotella**	-0.06	-0.46	-0.68	4.96E-01	-3.71	2.6E-04	-3.07	2.18E-03	2.59E-04
**Otu161_rc4-4**	0.39	0.49	3.51	5.31E-04	1.66	9.8E-02	3.62	2.94E-04	2.94E-04
**Otu101_Epulopiscium**	0.44	0.25	2.76	7.19E-03	2.43	1.6E-02	3.60	3.14E-04	3.14E-04
**Otu139_Desulfovibrio**	0.23	0.41	2.48	1.41E-02	2.64	8.9E-03	3.59	3.36E-04	3.36E-04
**Otu42_Actinobacillus**	0.23	0.24	2.61	1.01E-02	2.42	1.6E-02	3.52	4.31E-04	4.31E-04
**Otu5_Escherichia**	0.19	0.32	3.56	4.57E-04	1.23	2.2E-01	3.35	8.11E-04	4.57E-04
**Otu195_Pasteurellaceae**	0.38	0.26	2.55	1.27E-02	2.47	1.4E-02	3.49	4.75E-04	4.75E-04
**Otu591_Desulfovibrionaceae**	0.24	0.40	1.93	5.49E-02	3.05	2.5E-03	3.49	4.79E-04	4.79E-04
**Otu2402_Treponema**	-0.32	-0.24	-2.60	1.04E-02	-2.39	1.8E-02	-3.49	4.82E-04	4.82E-04
**Otu145_S24-7**	0.28	0.20	3.52	5.15E-04	0.96	3.4E-01	3.13	1.74E-03	5.15E-04
**Otu38_Lactobacillus**	-0.25	0.03	-3.52	5.25E-04	0.12	9.1E-01	2.37	1.79E-02	5.25E-04
**Otu645_Clostridiales**	-0.08	-0.36	-0.36	7.21E-01	-3.51	5.3E-04	-2.70	6.90E-03	5.34E-04
**Otu1423_Treponema**	-0.59	-0.13	-3.52	5.49E-04	-1.22	2.2E-01	-3.31	9.48E-04	5.49E-04
**Otu410_YS2**	0.52	0.22	2.93	4.51E-03	2.03	4.3E-02	3.44	5.91E-04	5.91E-04
**Otu575_Ruminococcaceae**	-0.17	-0.35	-0.96	3.41E-01	-3.48	5.9E-04	-3.10	1.92E-03	5.94E-04
**Otu1384_Ruminococcaceae**	0.57	0.08	3.50	5.99E-04	0.79	4.3E-01	2.99	2.81E-03	5.99E-04
**Otu208_CF231**	0.32	0.34	3.20	1.57E-03	1.69	9.2E-02	3.43	6.10E-04	6.10E-04

**Otu257_Prevotella**	0.31	0.23	2.60	1.04E-02	2.29	2.3E-02	3.42	6.28E-04	6.28E-04
**Otu171_Prevotella**	-0.26	-0.23	-3.05	2.62E-03	-1.77	7.7E-02	-3.38	7.32E-04	7.32E-04
**Otu649_Mogibacteriaceae**	0.17	0.33	0.73	4.69E-01	3.41	7.6E-04	2.89	3.81E-03	7.56E-04
**Otu1975_Treponema**	-0.31	-0.25	-2.35	2.00E-02	-2.45	1.5E-02	-3.36	7.69E-04	7.69E-04
**Otu2813_Treponema**	-0.14	-0.33	-1.04	3.00E-01	-3.40	7.9E-04	-3.10	1.91E-03	7.94E-04
**Otu2768_Clostridiaceae**	-0.15	-0.34	-1.02	3.07E-01	-3.38	8.3E-04	-3.08	2.04E-03	8.35E-04
**Otu2709_Prevotella**	0.22	0.30	2.62	9.35E-03	2.13	3.4E-02	3.33	8.54E-04	8.54E-04
**Otu468_Ruminococcus**	-0.06	-0.33	-0.54	5.90E-01	-3.36	9.0E-04	-2.73	6.35E-03	8.96E-04
**Otu118_Bacteroidales**	0.29	0.21	2.74	6.82E-03	1.99	4.8E-02	3.31	9.27E-04	9.27E-04
**Otu454_Mogibacteriaceae**	0.38	0.28	2.50	1.32E-02	2.20	2.8E-02	3.30	9.58E-04	9.58E-04
**Otu57_CF231**	0.33	-0.03	3.35	1.07E-03	-0.34	7.3E-01	-2.07	3.84E-02	1.07E-03
**Otu31_Treponema**	-0.28	-0.39	-3.31	1.07E-03	-1.00	3.2E-01	-3.02	2.53E-03	1.07E-03

Supplementary table 5: taxonomies significantly associated with blood glucose

**Taxonomy**	**Estimate_q**	**Estimate_b**	**T_value_q**	**P_value_q**	**T_value_b**	**P_value_b**	**meta_z_score**	**GLU_P_meta**	**final_p_value**
**g_Coprococcus**	1.69	0.11	6.11	4.11E-09	NA	NA	5.88	NA	4.11E-09
**g_Roseburia**	1.68	0.11	5.99	7.75E-09	NA	NA	5.77	NA	7.75E-09
**f_Lachnospiraceae/Lachnospira**	2.23	0.11	5.77	2.42E-08	NA	NA	5.58	NA	2.42E-08
**g_Faecalibacterium**	1.05	0.18	3.81	1.79E-04	0.49	0.62	3.75	2.74E-03	1.79E-04
**o_Turicibacterales/Turicibacteraceae/Turicibacter**	0.82	1.39	3.62	3.56E-04	1.59	0.11	3.64	2.70E-04	2.70E-04
**g_Peptococcus**	1.24	0.85	1.98	5.38E-02	3.14	0.00	3.56	3.75E-04	3.75E-04
**f_Enterococcaceae**	1.69	-1.39	1.43	1.72E-01	-3.31	0.00	3.27	1.78E-01	1.07E-03
**g_Clostridium**	0.06	0.78	0.06	9.55E-01	3.19	0.00	3.15	2.31E-02	1.61E-03
**g_Pseudobutyrivibrio**	0.63	0.67	1.38	1.70E-01	3.03	0.00	3.09	2.00E-03	2.00E-03
**g_Mogibacterium**	-1.30	-0.54	-2.08	4.12E-02	-2.29	0.02	-3.05	2.27E-03	2.27E-03
**o_Actinomycetales**	-0.93	-0.52	-2.18	3.14E-02	-2.32	0.02	-3.15	1.63E-03	1.63E-03

Taxonomy	Estimate_q	Estimate_b	T_value_q	P_value_q	T_value_b	P_value_b	meta_z_score	GLU_P_meta	final_p_value
**o_Bacillales**	-1.03	-0.80	-0.99	3.27E-01	-3.20	0.00	-3.17	3.38E-03	1.55E-03
**c_Actinobacteria**	-0.97	-0.50	-2.32	2.18E-02	-2.23	0.03	-3.19	1.42E-03	1.42E-03
**c_Deltaproteobacteria**	-0.80	-1.06	-3.66	3.10E-04	-1.20	0.23	-3.61	6.77E-04	3.10E-04
**f_Christensenellaceae**	-1.05	-0.63	-3.45	6.83E-04	-1.71	0.09	-3.61	3.07E-04	3.07E-04
**g_Fusobacterium**	-0.40	-0.55	-3.07	2.47E-03	-2.14	0.03	-3.64	2.68E-04	2.68E-04
**f_S24.7**	-1.50	0.11	-3.93	1.09E-04	NA	NA	-3.87	NA	1.09E-04
**f_Bacteroidaceae/Bacteroides**	-1.16	0.11	-4.04	7.22E-05	NA	NA	-3.97	NA	7.22E-05
**p_Fusobacteria/Fusobacteriia/Fusobacteriales/Fusobacteriaceae**	-0.56	-0.53	-4.72	4.37E-06	-1.61	0.11	-4.59	1.17E-05	4.37E-06
**o_Desulfovibrionales/Desulfovibrionaceae**	-1.20	-1.02	-4.75	3.51E-06	-1.62	0.11	-4.64	9.72E-06	3.51E-06
**f_Porphyromonadaceae**	-1.71	0.11	-4.77	3.20E-06	NA	NA	-4.66	NA	3.20E-06
**g_Parabacteroides**	-1.88	0.11	-4.93	1.56E-06	NA	NA	-4.80	NA	1.56E-06

Supplementary table 6: Taxonomies significantly associated with total cholesterol (TC)

taxonomy	Estimate_q	Estimate_b	T_value_q	P_value_q	T_value_b	P_value_b	meta_z_score	P_meta	final_p_value
**o_Lactobacillales/f_Lactobacillaceae/Lactobacillus**	-0.08	-0.66	-2.80	5.50E-03	-3.97	9.57E-05	-4.72	2.34E-06	2.34E-06
**f_BS11**	-0.06	-0.13	-2.08	3.95E-02	-2.88	4.32E-03	-3.47	5.14E-04	5.14E-04
**c_Bacilli**	-0.14	0.02	-3.49	5.84E-04	NA	NA	-3.44	NA	5.84E-04
**p_Fusobacteria/Fusobacteriia/Fusobacteriales/Fusobacteriaceae**	0.08	0.09	3.40	8.23E-04	1.43	1.55E-01	3.37	7.47E-04	7.47E-04
**g_Fusobacterium**	0.08	0.12	2.70	7.71E-03	2.40	1.71E-02	3.57	3.57E-04	3.57E-04
**g_Prevotella**	0.19	0.02	3.72	2.45E-04	NA	NA	3.67	NA	2.45E-04
**o_GMD14H09**	0.14	0.05	3.75	2.36E-04	0.86	3.93E-01	3.68	1.35E-03	2.36E-04
**c_Deltaproteobacteria**	0.19	0.05	4.49	1.11E-05	0.30	7.63E-01	4.39	8.98E-04	1.11E-05

Supplementary table 7: Taxonomies significantly associated with high density lipoprotein (HDL)

taxonomy	Estimate_q	Estimate_b	T_value_q	P_value_q	T_value_b	P_value_b	meta_z_score	P_meta	final_p_value
**f_Veillonellaceae**	-0.32	0.02	-4.51	1.01E-05	NA	NA	-4.42	NA	1.01E-05
**g_Phascolarctobacterium**	-0.28	0.02	-4.41	1.54E-05	NA	NA	-4.32	NA	1.54E-05
**p_Synergistetes/Synergistia/Synergistales**	-0.10	-0.19	-0.95	3.44E-01	-3.85	1.53E-04	-3.79	8.17E-04	1.53E-04
**f_Dethiosulfovibrionaceae**	-0.11	-0.19	-1.03	3.06E-01	-3.83	1.65E-04	-3.77	7.05E-04	1.65E-04
**g_CF231**	-0.24	0.02	-3.59	4.02E-04	NA	NA	-3.54	NA	4.02E-04
**g_Desulfovibrio**	-0.03	-0.21	-0.60	5.46E-01	-3.29	1.16E-03	-3.25	6.47E-03	1.16E-03
**c_Bacilli**	0.15	0.02	3.25	1.33E-03	NA	NA	3.21	NA	1.33E-03
**g_Anaeroplasma**	0.22	0.01	3.42	7.82E-04	0.25	8.03E-01	3.36	1.07E-02	7.82E-04
**g_Mitsuokella**	0.31	0.39	0.78	4.52E-01	3.55	4.72E-04	3.50	2.66E-03	4.72E-04
**o_Lactobacillales/f_Lactobacillaceae/Lactobacillus**	0.12	0.10	3.66	3.07E-04	0.50	6.14E-01	3.61	3.63E-03	3.07E-04

Supplementary table 8: Taxonomies significantly associated with low density lipoprotein (LDL)

taxonomy	Estimate_q	Estimate_b	T_value_q	P_value_q	T_value_b	P_value_b	meta_z_score	P_meta	final_p_value
**c_Deltaproteobacteria**	0.17	-0.08	4.60	6.91E-06	-0.54	5.88E-01	-4.50	5.16E-03	6.91E-06
**f_Enterococcaceae**	-0.18	0.31	-0.41	6.86E-01	4.50	1.06E-05	-4.40	4.68E-03	1.06E-05
**g_Vagococcus**	-0.35	0.35	-0.33	7.45E-01	4.14	4.74E-05	-4.07	8.14E-03	4.74E-05
**o_Desulfovibrionales/Desulfovibrionaceae**	0.17	-0.01	4.03	7.63E-05	-0.11	9.12E-01	-3.96	6.55E-03	7.63E-05
**c_Gammaproteobacteria**	0.08	-0.02	3.66	3.07E-04	-0.08	9.40E-01	-3.61	1.25E-02	3.07E-04
**p_Spirochaetes/Spirochaetes/Spirochaetales/Spirochaetaceae/Treponema**	-0.09	0.02	-3.25	1.31E-03	NA	NA	-3.21	NA	1.31E-03
**o_GMD14H09**	0.11	-0.01	3.20	1.61E-03	-0.14	8.87E-01	-3.15	3.33E-02	1.61E-03
**g_Mitsuokella**	0.40	0.30	0.65	5.30E-01	3.55	4.60E-04	3.50	3.48E-03	4.60E-04
**f_Enterobacteriaceae/Enterobacteriales/Escherichia**	0.08	0.11	3.77	2.07E-04	1.52	1.29E-01	3.71	2.18E-04	2.07E-04
**p_Proteobacteria**	0.16	0.02	3.92	1.18E-04	NA	NA	3.85	NA	1.18E-04
**g_Fusobacterium**	0.09	0.13	3.29	1.20E-03	2.98	3.14E-03	4.38	1.19E-05	1.19E-05

**f_Aeromonadaceae**	0.05	0.29	0.28	7.81E-01	4.84	2.33E-06	4.72	4.06E-04	2.33E-06
**p_Fusobacteria/Fusobacteriia/Fusobacteriales/Fusobacteriaceae**	0.10	0.09	5.13	6.82E-07	1.64	1.02E-01	4.97	3.02E-06	6.82E-07

Supplementary table 9: Taxonomies significantly associated with ratio of low density lipoprotein to high density lipoprotein (LDL/HDL)

taxonomy	Estimate_q	Estimate_b	T_value_q	P_value_q	T_value_b	P_value_b	meta_z_score	P_meta	final_p_value
**f_Aeromonadaceae**	-0.06	0.54	-0.17	8.64E-01	4.41	1.56E-05	-4.32	3.35E-03	1.56E-05
**g_Roseburia**	-0.43	0.04	-4.35	2.01E-05	NA	NA	-4.26	NA	2.01E-05
**g_Anaeroplasma**	-0.36	-0.06	-3.72	2.71E-04	-0.72	4.71E-01	-3.64	2.04E-03	2.71E-04
**c_Bacilli**	-0.25	0.04	-3.49	5.78E-04	NA	NA	-3.44	NA	5.78E-04
**o_Turicibacterales/Turicibacteraceae/Turicibacter**	-0.26	-0.24	-3.39	8.31E-04	-0.80	4.25E-01	-3.34	3.42E-03	8.31E-04
**o_Anaeroplasmatales/Anaeroplasmataceae**	-0.33	-0.09	-3.37	9.23E-04	-1.09	2.78E-01	-3.31	1.88E-03	9.23E-04
**f_Lactobacillaceae/Lactobacillus**	-0.17	-0.31	-3.27	1.25E-03	-1.04	2.98E-01	-3.23	2.54E-03	1.25E-03
**o_Spirochaetales/Spirochaetaceae/Treponema**	-0.17	0.04	-3.16	1.76E-03	NA	NA	-3.13	NA	1.76E-03
**g_Coprococcus**	-0.31	0.04	-3.08	2.29E-03	NA	NA	-3.05	NA	2.29E-03
**f_Paraprevotellaceae**	0.41	0.04	3.08	2.33E-03	NA	NA	3.04	NA	2.33E-03
**f_Porphyromonadaceae**	0.39	0.04	3.13	1.95E-03	NA	NA	3.10	NA	1.95E-03
**g_Desulfovibrio**	0.21	0.20	2.54	1.20E-02	1.90	5.82E-02	3.12	1.83E-03	1.83E-03
**p_Proteobacteria**	0.27	0.04	3.18	1.69E-03	NA	NA	3.14	NA	1.69E-03
**g_Actinobacillus**	0.17	0.21	2.08	4.00E-02	2.78	5.84E-03	3.40	6.71E-04	6.71E-04
**c_Gammaproteobacteria**	0.16	0.43	3.53	5.01E-04	1.00	3.17E-01	3.48	1.53E-03	5.01E-04
**g_rc4.4**	0.32	0.30	3.19	1.65E-03	1.89	6.06E-02	3.55	3.81E-04	3.81E-04
**f_Mogibacteriaceae**	0.37	0.04	3.64	3.39E-04	NA	NA	3.58	NA	3.39E-04
**o_Pasteurellales/Pasteurellaceae**	0.19	0.20	2.60	1.05E-02	2.56	1.11E-02	3.61	3.10E-04	3.10E-04
**g_Epulopiscium**	0.06	-0.45	0.73	4.65E-01	-3.72	2.50E-04	3.66	3.82E-02	2.50E-04
**p_Synergistetes/Synergistia/Synergistales**	0.29	0.29	1.49	1.37E-01	3.81	1.77E-04	3.75	2.13E-04	1.77E-04
**f_Peptococcaceae**	0.33	0.44	3.48	6.10E-04	1.92	5.62E-02	3.77	1.61E-04	1.61E-04

**c_Deltaproteobacteria/Dethiosulfovibrionaceae**	0.29	0.01	3.95	1.02E-04	0.04	9.71E-01	3.89	5.55E-03	1.02E-04
**f_Dethiosulfovibrionaceae**									
**o_Desulfovibrionales/Desulfovibrionaceae**	0.35	0.09	4.03	7.60E-05	0.41	6.83E-01	3.96	2.02E-03	7.60E-05
**f_Enterobacteriaceae/Enterobacteriales**	0.16	0.34	3.45	6.70E-04	2.35	1.96E-02	4.06	5.00E-05	5.00E-05
**g_CF231**	0.42	0.04	4.14	4.78E-05	NA	NA	4.07	NA	4.78E-05
**f_Veillonellaceae**	0.47	0.04	4.14	4.74E-05	NA	NA	4.07	NA	4.74E-05
**p_Fusobacteria/Fusobacteriia/Fusobacteriales/Fusobacteriaceae**	0.15	0.28	3.59	4.19E-04	2.51	1.26E-02	4.26	2.06E-05	2.06E-05
**g_Phascolarctobacterium**	0.44	0.04	4.39	1.70E-05	NA	NA	4.30	NA	1.70E-05

Supplementary table 10: Taxonomies significantly associated with atherosclerosis index (AI)

taxonomy	Estimate_q	Estimate_b	T_value_q	p_value_q	T_value_b	p_value_b	meta_z_score	p_meta	final_p_value
**c_Bacilli**	-0.41	0.05	-4.66	5.17E-06	NA	NA	-4.56	NA	5.17E-06
**o_Lactobacillales/f_Lactobacillaceae/Lactobacillus**	-0.31	-0.57	-4.65	5.51E-06	-1.469	1.43E-01	-4.54	2.15E-05	5.51E-06
**g_Roseburia**	-0.52	0.05	-4.10	5.62E-05	NA	NA	-4.03	NA	5.62E-05
**f_Aeromonadaceae**	-0.09	0.60	-0.26	8.00E-01	3.802	1.82E-04	-3.74	1.36E-02	1.82E-04
**g_Epulopiscium**	-0.01	-0.54	-0.06	9.50E-01	-3.489	5.77E-04	-3.44	1.32E-02	5.77E-04
**f_Clostridiaceae**	-0.47	0.05	-3.49	5.77E-04	NA	NA	-3.44	NA	5.77E-04
**g_Anaeroplasma**	-0.46	-0.07	-3.49	6.22E-04	-0.615	5.39E-01	-3.42	4.32E-03	6.22E-04
**o_Turicibacterales/Turicibacteraceae/Turicibacter**	-0.33	-0.33	-3.30	1.11E-03	-0.853	3.95E-01	-3.26	3.63E-03	1.11E-03
**o_Spirochaetales/Spirochaetaceae/Treponema**	-0.22	0.05	-3.16	1.76E-03	NA	NA	-3.13	NA	1.76E-03
**o_Anaeroplasmatales/Anaeroplasmataceae**	-0.40	-0.09	-3.11	2.18E-03	-0.826	4.10E-01	-3.06	5.95E-03	2.18E-03
**f_Mogibacteriaceae**	0.37	0.05	2.82	5.21E-03	NA	NA	2.79	NA	5.21E-03
**p_Proteobacteria**	0.31	0.05	2.88	4.29E-03	NA	NA	2.86	NA	4.29E-03
**f_Porphyromonadaceae**	0.49	0.05	3.01	2.85E-03	NA	NA	2.98	NA	2.85E-03
**o_Desulfovibrionales/Desulfovibrionaceae**	0.38	0.26	3.33	9.95E-04	0.944	3.46E-01	3.29	2.75E-03	9.95E-04
**c_Gammaproteobacteria**	0.19	0.83	3.17	1.72E-03	1.537	1.26E-01	3.30	9.69E-04	9.69E-04

**g_Actinobacillus**	0.18	0.28	1.85	6.63E-02	2.910	3.96E-03	3.34	8.51E-04	8.51E-04
**g_Desulfovibrio**	0.24	0.36	2.15	3.25E-02	2.772	6.01E-03	3.45	5.51E-04	5.51E-04
**g_rc4.4**	0.40	0.40	3.07	2.43E-03	1.960	5.12E-02	3.52	4.27E-04	4.27E-04
**o_Pasteurellales/Pasteurellaceae**	0.23	0.26	2.44	1.62E-02	2.626	9.21E-03	3.54	3.98E-04	3.98E-04
**f_Paraprevotellaceae**	0.62	0.05	3.63	3.45E-04	NA	NA	3.58	NA	3.45E-04
**c_Deltaproteobacteria**	0.35	0.26	3.72	2.50E-04	0.678	4.99E-01	3.66	2.15E-03	2.50E-04
**o_Enterobacteriales/Enterobacteriaceae/Escherichia**	0.19	0.38	3.26	1.31E-03	2.043	4.22E-02	3.71	2.08E-04	2.08E-04
**p_Fusobacteria/Fusobacteriia/Fusobacteriales/Fusobacteriaceae**	0.16	0.34	2.99	3.17E-03	2.395	1.74E-02	3.77	1.64E-04	1.64E-04
**f_Peptococcaceae**	0.46	0.57	3.78	1.97E-04	1.965	5.06E-02	4.01	5.95E-05	5.95E-05
**g_CF231**	0.57	0.05	4.43	1.46E-05	NA	NA	4.33	NA	1.46E-05
**f_Dethiosulfovibrionaceae**	0.39	0.44	1.67	9.78E-02	4.507	1.03E-05	4.41	1.79E-05	1.03E-05
**p_Synergistetes/Synergistia/Synergistales**	0.36	0.44	1.53	1.27E-01	4.522	9.65E-06	4.42	2.59E-05	9.65E-06
**f_Veillonellaceae**	0.68	0.05	4.81	2.65E-06	NA	NA	4.70	NA	2.65E-06
**g_Phascolarctobacterium**	0.63	0.05	4.99	1.14E-06	NA	NA	4.87	NA	1.14E-06

Supplementary table 11. KEGG functional pathway significantly associated with blood glucose.

LEVEL1	LEVEL2	LEVEL3	Estimate	T_value	z_score	p_value
**Metabolism**	Energy Metabolism	Carbon fixation pathways in prokaryotes	-20.09	-5.75	-5.56	2.71E-08
**Metabolism**	Energy Metabolism	Nitrogen metabolism	-31.39	-5.74	-5.55	2.89E-08
**Metabolism**	Biosynthesis of Other Secondary Metabolites	Tropane, piperidine and pyridine alkaloid biosynthesis	-21.48	-5.44	-5.27	1.34E-07
**Metabolism**	Amino Acid Metabolism	Cysteine and methionine metabolism	-70.29	-5.36	-5.20	2.00E-07
**Environmental Information Processing**	Signal Transduction	MAPK signaling pathway - yeast	-11.57	-5.31	-5.16	2.53E-07
**Metabolism**	Metabolism of Other Amino Acids	D-Glutamine and D-glutamate metabolism	-29.77	-5.29	-5.14	2.71E-07
**Metabolism**	Xenobiotics Biodegradation and Metabolism	Ethylbenzene degradation	-11.45	-5.03	-4.90	9.53E-07
**Metabolism**	Carbohydrate Metabolism	Citrate cycle (TCA cycle)	-10.37	-4.92	-4.79	1.65E-06
**Metabolism**	Nucleotide Metabolism	Purine metabolism	-39.82	-4.69	-4.58	4.54E-06

**Metabolism**	Biosynthesis of Other Secondary Metabolites	Isoquinoline alkaloid biosynthesis	-7.48	-4.64	-4.54	5.72E-06
**Metabolism**	Biosynthesis of Other Secondary Metabolites	Novobiocin biosynthesis	-22.49	-4.61	-4.50	6.69E-06
**Metabolism**	Metabolism of Cofactors and Vitamins	Folate biosynthesis	-11.34	-4.53	-4.44	9.15E-06
**Metabolism**	Xenobiotics Biodegradation and Metabolism	Naphthalene degradation	-9.80	-4.49	-4.40	1.09E-05
**Environmental Information Processing**	Signaling Molecules and Interaction	Cellular antigens	-3.28	-4.33	-4.25	2.16E-05
**Metabolism**	Xenobiotics Biodegradation and Metabolism	Toluene degradation	-4.54	-4.22	-4.14	3.51E-05
**Metabolism**	Amino Acid Metabolism	Tyrosine metabolism	-17.09	-4.19	-4.11	3.96E-05
**Metabolism**	Glycan Biosynthesis and Metabolism	Lipopolysaccharide biosynthesis proteins	-4.03	-4.09	-4.02	5.88E-05
**Organismal Systems**	Digestive System	Protein digestion and absorption	-2.40	-4.01	-3.94	8.13E-05
**Metabolism**	Glycan Biosynthesis and Metabolism	Lipopolysaccharide biosynthesis	-3.17	-4.00	-3.93	8.39E-05
**Metabolism**	Metabolism of Terpenoids and Polyketides	Prenyltransferases	-13.28	-3.98	-3.91	9.11E-05
**Metabolism**	Metabolism of Other Amino Acids	Taurine and hypotaurine metabolism	-14.02	-3.96	-3.90	9.74E-05
**Metabolism**	Amino Acid Metabolism	Glycine, serine and threonine metabolism	-33.63	-3.95	-3.88	1.03E-04
**Metabolism**	Metabolism of Cofactors and Vitamins	Nicotinate and nicotinamide metabolism	-18.05	-3.94	-3.87	1.09E-04
**Metabolism**	Glycan Biosynthesis and Metabolism	Glycosphingolipid biosynthesis - ganglio series	-3.13	-3.92	-3.86	1.14E-04
**Metabolism**	Metabolism of Cofactors and Vitamins	Riboflavin metabolism	-10.47	-3.89	-3.83	1.29E-04
**Metabolism**	Carbohydrate Metabolism	Butanoate metabolism	-17.05	-3.79	-3.73	1.94E-04
**Environmental Information Processing**	Membrane Transport	Bacterial secretion system	-16.45	-3.70	-3.65	2.63E-04
**Metabolism**	Enzyme Families	Peptidases	-34.45	-3.69	-3.63	2.80E-04
**Metabolism**	Glycan Biosynthesis and Metabolism	N-Glycan biosynthesis	-4.06	-3.67	-3.62	2.97E-04
**Metabolism**	Metabolism of Cofactors and Vitamins	Ubiquinone and other terpenoid-quinone biosynthesis	-3.79	-3.65	-3.60	3.19E-04
**Cellular Processes**	Transport and Catabolism	Peroxisome	-8.84	-3.65	-3.59	3.26E-04
**Metabolism**	Amino Acid Metabolism	Alanine, aspartate and glutamate metabolism	-21.73	-3.61	-3.56	3.77E-04
**Metabolism**	Glycan Biosynthesis and Metabolism	Glycosaminoglycan degradation	-3.61	-3.59	-3.54	3.99E-04
**Metabolism**	Lipid Metabolism	Arachidonic acid metabolism	-3.05	-3.57	-3.52	4.24E-04
**Metabolism**	Metabolism of Terpenoids and Polyketides	Polyketide sugar unit biosynthesis	-12.97	-3.55	-3.50	4.58E-04

**Metabolism**	Glycan Biosynthesis and Metabolism	Glycosyltransferases	-10.87	-3.49	-3.44	5.74E-04
**Metabolism**	Metabolism of Cofactors and Vitamins	Biotin metabolism	-8.77	-3.44	-3.39	6.89E-04
**Metabolism**	Metabolism of Cofactors and Vitamins	Retinol metabolism	-3.95	-3.32	-3.27	1.06E-03
**Metabolism**	Nucleotide Metabolism	Pyrimidine metabolism	-27.69	-3.23	-3.19	1.44E-03
**Metabolism**	Metabolism of Other Amino Acids	Glutathione metabolism	-6.75	-3.21	-3.17	1.53E-03
**Organismal Systems**	Endocrine System	Adipocytokine signaling pathway	-4.15	-3.16	-3.13	1.76E-03
**Metabolism**	Metabolism of Cofactors and Vitamins	One carbon pool by folate	-14.57	-3.11	-3.08	2.07E-03
**Metabolism**	Amino Acid Metabolism	Phenylalanine metabolism	-12.20	-3.07	-3.04	2.40E-03
**Metabolism**	Biosynthesis of Other Secondary Metabolites	Streptomycin biosynthesis	-13.32	-3.02	-2.99	2.82E-03
**Metabolism**	Metabolism of Terpenoids and Polyketides	Biosynthesis of siderophore group nonribosomal peptides	-2.95	-2.99	-2.96	3.12E-03
**Metabolism**	Biosynthesis of Other Secondary Metabolites	Penicillin and cephalosporin biosynthesis	-3.53	-2.89	-2.86	4.19E-03
**Metabolism**	Biosynthesis of Other Secondary Metabolites	Stilbenoid, diarylheptanoid and gingerol biosynthesis	-0.43	-1.65	-2.83	4.64E-03
**Environmental Information Processing**	Signal Transduction	Phosphatidylinositol signaling system	-8.28	-2.84	-2.82	4.85E-03
**Metabolism**	Glycan Biosynthesis and Metabolism	Peptidoglycan biosynthesis	-21.48	-2.83	-2.80	5.05E-03
**Metabolism**	Metabolism of Other Amino Acids	beta-Alanine metabolism	-7.37	-2.78	-2.75	5.90E-03
**Metabolism**	Metabolism of Cofactors and Vitamins	Vitamin B6 metabolism	-10.32	-2.77	-2.74	6.14E-03
**Metabolism**	Xenobiotics Biodegradation and Metabolism	Drug metabolism - other enzymes	-15.31	-2.76	-2.73	6.26E-03
**Metabolism**	Metabolism of Terpenoids and Polyketides	Terpenoid backbone biosynthesis	-18.14	-2.70	-2.68	7.40E-03
**Metabolism**	Amino Acid Metabolism	Valine, leucine and isoleucine degradation	-4.66	-2.69	-2.66	7.73E-03
**Metabolism**	Metabolism of Terpenoids and Polyketides	Geraniol degradation	-2.09	-2.68	-2.66	7.85E-03
**Metabolism**	Energy Metabolism	Oxidative phosphorylation	-10.49	-2.67	-2.65	8.04E-03
**Metabolism**	Lipid Metabolism	Fatty acid metabolism	-5.54	-2.66	-2.64	8.37E-03
**Metabolism**	Xenobiotics Biodegradation and Metabolism	Aminobenzoate degradation	-6.85	-2.64	-2.61	8.94E-03
**Metabolism**	Metabolism of Other Amino Acids	Selenocompound metabolism	-20.28	-2.59	-2.57	1.01E-02
**Metabolism**	Metabolism of Cofactors and Vitamins	Lipoic acid metabolism	-2.90	-2.59	-2.57	1.02E-02
**Metabolism**	Lipid Metabolism	Steroid hormone biosynthesis	-1.21	-2.55	-2.53	1.14E-02

**Metabolism**	Metabolism of Terpenoids and Polyketides	Biosynthesis of vancomycin group antibiotics	-8.42	-2.50	-2.48	1.30E-02
**Metabolism**	Biosynthesis of Other Secondary Metabolites	Isoflavonoid biosynthesis	-0.81	-2.12	-2.47	1.37E-02
**Organismal Systems**	Excretory System	Proximal tubule bicarbonate reclamation	-2.84	-2.47	-2.45	1.42E-02
**Metabolism**	Carbohydrate Metabolism	Inositol phosphate metabolism	-5.45	-2.47	-2.45	1.43E-02
**Cellular Processes**	Transport and Catabolism	Lysosome	-2.78	-2.28	-2.27	2.32E-02
**Metabolism**	Carbohydrate Metabolism	Propanoate metabolism	-11.07	-2.27	-2.26	2.40E-02
**Metabolism**	Lipid Metabolism	Steroid biosynthesis	-0.71	-2.31	1.38	2.20E-02
**Metabolism**	Carbohydrate Metabolism	C5-Branched dibasic acid metabolism	16.42	2.49	2.47	1.36E-02
**Environmental Information Processing**	Signal Transduction	Two-component system	11.38	2.55	2.53	1.14E-02
**Metabolism**	Biosynthesis of Other Secondary Metabolites	Butirosin and neomycin biosynthesis	6.88	2.63	2.60	9.20E-03
**Metabolism**	Xenobiotics Biodegradation and Metabolism	Bisphenol degradation	12.37	2.64	2.61	8.96E-03
**Metabolism**	Enzyme Families	Protein kinases	9.13	2.67	2.65	8.12E-03
**Metabolism**	Carbohydrate Metabolism	Galactose metabolism	11.17	2.69	2.67	7.58E-03
**Metabolism**	Lipid Metabolism	Glycerolipid metabolism	10.02	2.82	2.79	5.24E-03
**Metabolism**	Amino Acid Metabolism	Valine, leucine and isoleucine biosynthesis	18.32	2.85	2.82	4.79E-03
**Metabolism**	Energy Metabolism	Photosynthesis	11.00	3.03	3.00	2.73E-03
**Metabolism**	Energy Metabolism	Photosynthesis proteins	11.43	3.12	3.09	2.01E-03
**Metabolism**	Carbohydrate Metabolism	Starch and sucrose metabolism	14.81	3.53	3.48	5.05E-04
**Cellular Processes**	Cell Motility	Cytoskeleton proteins	11.21	3.56	3.51	4.48E-04
**Metabolism**	Carbohydrate Metabolism	Fructose and mannose metabolism	20.46	3.72	3.66	2.48E-04
**Organismal Systems**	Endocrine System	Insulin signaling pathway	10.16	3.76	3.70	2.15E-04
**Metabolism**	Carbohydrate Metabolism	Pentose and glucuronate interconversions	15.93	3.77	3.71	2.06E-04
**Metabolism**	Lipid Metabolism	Primary bile acid biosynthesis	6.09	3.88	3.82	1.36E-04
**Environmental Information Processing**	Membrane Transport	ABC transporters	10.94	3.97	3.90	9.49E-05
**Metabolism**	Lipid Metabolism	Secondary bile acid biosynthesis	6.23	4.15	4.07	4.66E-05
**Cellular Processes**	Cell Motility	Bacterial chemotaxis	5.67	4.44	4.34	1.39E-05

**Metabolism**	Metabolism of Terpenoids and Polyketides	Biosynthesis of ansamycins	11.07	4.45	4.36	1.30E-05
**Metabolism**	Metabolism of Other Amino Acids	D-Arginine and D-ornithine metabolism	2.77	4.52	4.42	9.65E-06
**Environmental Information Processing**	Membrane Transport	Transporters	10.98	4.60	4.50	6.73E-06
**Cellular Processes**	Cell Motility	Bacterial motility proteins	7.09	4.64	4.53	5.76E-06
**Cellular Processes**	Cell Motility	Flagellar assembly	6.14	4.80	4.69	2.77E-06
**Metabolism**	Carbohydrate Metabolism	Pentose phosphate pathway	25.41	5.07	4.94	7.88E-07
**Organismal Systems**	Environmental Adaptation	Plant-pathogen interaction	21.32	5.15	5.01	5.47E-07
